# Multidimensional Engineering of *Escherichia coli* for Efficient Adipic Acid Synthesis From Cyclohexane

**DOI:** 10.1002/advs.202411938

**Published:** 2025-02-17

**Authors:** Fei Wang, Huiqi Sun, Di Deng, Yuanqing Wu, Jing Zhao, Qian Li, Aitao Li

**Affiliations:** ^1^ State Key Laboratory of Biocatalysis and Enzyme Engineering Hubei Key Laboratory of Industrial Biotechnology School of Life Sciences Hubei University Wuhan 430062 P. R. China; ^2^ School of Synthetic Biology Shanxi University Taiyuan 030031 P. R. China

**Keywords:** adipic acid, balance, biosynthesis, cascade pathway

## Abstract

Adipic acid (AA), a key aliphatic dicarboxylic acid, is conventionally manufactured through energy‐intensive, multi‐step chemical processes with significant environmental impacts. In contrast, biological production methods offer more sustainable alternatives but are often limited by low productivity. To overcome these challenges, this study reports the engineering of a single *Escherichia coli* for efficient biosynthesis of AA starting from cyclohexanol (CHOL), KA oil (mixture of CHOL and cyclohexanone (CHONE)), or cyclohexane (CH). To start with, a comprehensive screening of rate‐limiting enzymes is conducted, particularly focusing on cytochrome P450 monooxygenase, followed by the optimization of protein expression using strategies such as protein fusion, promoter replacement, and genome editing. Consequently, an engineered *E. coli* capable of efficiently converting either KA oil or CH into AA is obtained, achieving remarkable product titers of 110 and 22.6 g L^−1^, respectively. This represents the highest productivity record for the biological production of AA to date. Finally, this developed biocatalytic system is successfully employed to convert different cycloalkanes and cycloalkanols with varied carbon chain lengths into their corresponding dicarboxylic acids, highlighting its great potential as well as broad applicability for industrial applications.

## Graphical abstract

An engineered *Escherichia coli* is developed to efficiently produce adipic acid (AA) from cyclohexanol, KA oil (a mixture of cyclohexanol and cyclohexanone), or cyclohexane through a combination of enzyme screening, protein fusion, and genome editing techniques. This approach has achieved the highest recorded productivity for biological AA production to date, showcasing significant potential as an alternative to traditional chemical processes.

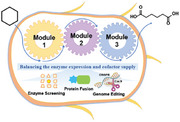

## 1 Introduction

Adipic acid (AA), identified by the International Energy Agency as the most important aliphatic dicarboxylic acid, holds significant industrial production potential.^[^ ^1^ ^]^ Predominantly, AA is utilized as a key monomer in the synthesis of nylon 6,6 paired with hexanediamine (HMD). Beyond its role in nylon production, AA is extensively used in the manufacturing of polyesters, polyurethanes, lubricants, and plasticizers. Intriguingly, it also serves as a gelling agent for flavorings in the food industry.^[^ ^2^ ^]^ With an annual global production of ≈3 million tons, the market for AA is anticipated to exceed $8 billion by 2025.^[^ ^3^ ^]^

However, the dominant method for AA production at present is heavily dependent on nitric acid oxidation of KA‐oil, a mixture of cyclohexanone (CHONE) and cyclohexanol (CHOL) obtained from cyclohexane (CH).^[^ ^2^, ^4^, ^5^, ^6^ ^]^ This process is both energy‐intensive and environmentally detrimental, generating nitrous oxide (N_2_O) emissions with a global warming potential 300‐fold higher than that of carbon dioxide (CO_2_).^[^ ^7^, ^8^ ^]^ Moreover, it generates by‐products like succinic acid and glutaric acid, which makes the purification of the final product more challenging.^[^ ^9^ ^]^ Therefore, a variety of alternative synthetic methods have been proposed and developed to address environmental issues as well as to enhance the catalytic efficiency.

To date, several nitric acid‐free methods for the synthesis of AA have been developed. These include the oxidation of cyclohexene using hydrogen peroxide^[^ ^5^, ^10^ ^]^ and double carbonylation of butadiene catalyzed by heavy metal catalysts such as cobalt, palladium, and rhodium.^[^ ^11^ ^]^ Despite the significant strides made, these methods still face challenges related to harsh reaction conditions and issues with catalyst stability, preparation, and catalyst recovery.^[^ ^11^ ^]^

On the other hand, metabolic engineering has significantly advanced AA production through environmentally friendly biological processes. For instance, a genetically modified *E. coli* expressing a five‐step reverse adipate‐degradation pathway (RADP) was developed, with further metabolic pathway optimization to enhance the precursor accumulation and reduce the competing metabolites. This engineered *E. coli* achieved an impressive production level of 68.0 g L^−1^ of AA through fed‐batch fermentation using glycerol as the carbon source.^[^ ^12^ ^]^ Additionally, engineered *C. tropicalis* successfully produced AA from long‐chain carbon substrates through successive ω‐ and β‐oxidation of methyl laurate, resulting in a production level of ca. 12.1 g L^−1^.^[^ ^13^ ^]^ Furthermore, *cis, cis*‐muconic acid was first biosynthesized from glucose via the shikimate pathway in engineered *Saccharomyces cerevisiae*, which was subsequently converted to AA using enoate reductases (ER). However, only 2.59 mg L^−1^ AA was produced during this process.^[^ ^14^, ^15^ ^]^

Nevertheless, it needs to be pointed out that all the aforementioned routes require careful selection of appropriate host organisms and optimization of metabolic pathways. Moreover, these pathways are often governed by inherent regulatory mechanisms that necessitate complex genetic manipulations to establish an efficient biomanufacturing process. In contrast to endogenous metabolic pathway engineering, which typically begins with glucose or glycerol as starting material, in vivo, multi‐enzyme cascade biosynthesis offers a more flexible approach for designing and constructing artificial synthetic pathways tailored to specific requirements. This approach has been utilized to produce AA from catechol or guaiacol in engineered *E. coli*, but it has yielded only ca. 0.6 g L^−1^ AA.^[^ ^16^ ^]^ Similarly, genetically engineered *Pseudomonas taiwanensis* demonstrated the ability to convert CH to AA, achieving a production level of 10.2 g L^−1^.^[^ ^17^ ^]^ However, this still falls short of industrial demands. In our previous work, we successfully utilized a microbial consortium of three different *E. coli* cells for AA synthesis from CH. However, the implementation of the consortium system introduced further complexities at both the cultivation and reaction stages, resulting in a product concentration of only 4.5 g L^−1^, which is also insufficient for industrial applications.^[^ ^18^ ^]^

**Figure 1:**
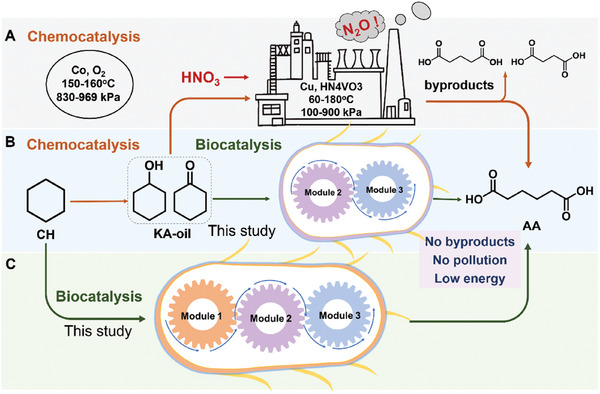
Industrial chemical and proposed biocatalytic processes for AA production. A) Current chemical industrial process for the synthesis of AA. B) Proposed biosynthesis route for AA production from KA oil. C) Designed biosynthesis route for AA production by a single engineered *E. coli* harboring corresponding enzymes.

In this study, we aimed to engineer a single *E. coli* that incorporates a meticulously designed cascade pathway to significantly enhance AA production using CHOL (or KA oil) and CH as readily available substrates (**Figure** **1**). This process employs a bottom‐up design approach, enabling the creation of a flexible and efficient biosynthetic route. It involves rigorous screening of rate‐limiting enzymes and employs techniques such as protein fusion, gene insertions, gene knockouts, and promoter substitution to balance the enzyme expression and cofactor supply. Subsequently, we established this single *E. coli*‐based biocatalytic system for efficient conversion of simple and readily available substrates (KA oil or CH) to AA. Finally, we demonstrated the scalability and generality of this developed catalytic system through scaled‐up reactions in a bioreactor and testing its ability to convert various cycloalkanes and cycloalkanols with varied carbon chain lengths into their corresponding dicarboxylic acids (DCAs).

## 2 Results

### 2.1 Design of Single *E. coli* Cell for Converting CH to AA

In our previous study, we successfully constructed *E. coli* consortia for the synthesis of AA from CH.^[^ ^18^ ^]^ However, this process was labor‐intensive, time‐consuming, and yielded low productivity of AA (33 mm or 4.5 g L^−1^), which is far from meeting the demands of industrialization. To streamline the process, we aimed to develop a single *E. coli* cell capable of converting CH to AA. However, in our initial attempt, coexpressing all enzymes in *E. coli* only led to the formation of trace amounts of AA (2–4 mm), likely due to the imbalances of protein expression or/and insufficient activity of the critical rate‐limiting enzymes.^[^ ^18^ ^]^ To address these issues, the following strategies are proposed: 1) enhancing the activity of key rate‐limiting enzymes by identifying the most suitable candidates, 2) balancing protein expression through genome integration, protein fusion, and promoter optimization, and 3) fine‐tuning cellular cofactor supply through gene knockout and promoter replacement. To implement these strategies, a bottom‐up reverse engineering approach was adopted, starting with the target product AA and systematically working backward. Specifically, ε‐caprolactone (ε‐CL), CHOL, and CH were used as substrates to progressively expand the biocatalytic pathway within an engineered *E. coli*, aiming to create a more efficient and streamlined biosynthetic process.

**Figure 2:**
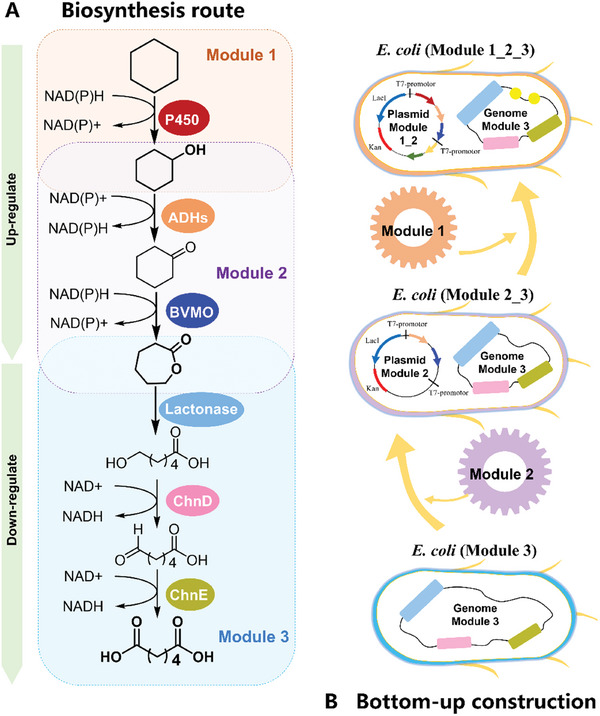
Concept of Single *E. coli* construction for converting CH to AA with a bottom‐up approach. A) The biosynthesis route from CH to AA contains three basic modules respectively. Module 1(orange block): P450‐catalyzed hydroxylation of CH to CHOL; Module 2 (purple block): Alcohol dehydrogenase (ADH) and Baeyer‐Villiger monooxygenase (BVMO) for two‐step oxidation of CHOL to ε‐CL; Module 3 (blue block): Lactonase for hydrolysis of ε‐CL to 6‐HHA and alcohol dehydrogenase (ChnD) and aldehyde dehydrogenase (ChnE) for two sequential oxidation steps of 6‐HHA to AA. The expression of enzymes in Module 1 and Module 2 is upregulated, whereas the expression of enzymes in Module 3 is downregulated. B) Bottom‐up reverse strategy for the construction of single *E. coli* for converting CHOL or CH to produce AA. First, *E. coli* (Module 3) was constructed to produce AA from ε‐CL. Next, optimal enzymes in Module 2 were integrated to create *E. coli* (Module 2_3) for the biotransformation from CHOL to AA. Subsequently, selected enzymes in Module 1 were incorporated into *E. coli* (Module 2_3) with the enhancement of cofactor (NADPH/NADH) supply. Finally, the recombinant single *E. coli* (Module 1_2_3) capable of converting CH to AA was obtained.

As depicted in **Figure** **2**, the biocatalytic cascade was divided into three modules, Module 1 involves the conversion of CH to CHOL; Module 2 focuses on the transformation of CHOL to ε‐CL; and Module 3 is responsible for the production of AA from ε‐CL. The first step in our approach is the construction of a recombinant *E. coli* (designated as *E. coli* (Module 3)) that expresses lactonase, alcohol dehydrogenas (ChnD), and aldehyde dehydrogenase (ChnE), which are essential for the biotransformation of ε‐CL to AA. Given that the enzymes in Module 3 exhibit better catalytic performance than those in other modules,^[^ ^18^ ^]^ the relevant genes were integrated into the *E. coli* genome to achieve lower expression levels while still ensuring sufficient catalytic activity for the desired reaction. Subsequently, enzymes from Module 2, which convert CHOL to ε‐CL, were further introduced to generate a recombinant *E. coli* (named *E. coli* (Module 2_3)) capable of converting CHOL to AA. Due to the relatively lower catalytic activity of the enzymes in Module 2, strategies including screening for optimal alcohol dehydrogenase (ADH), tag fusion to boost the expression of Baeyer‐Villiger monooxygenase (BVMO), and the use of high‐copy‐number plasmids with a strong P*_T7_* promoter for improving their expression, were employed. Moreover, to reduce the accumulation of intermediate 6‐hydroxyhexanoic acid (6‐HHA), promoter optimization for ChnD expression in *E. coli* (Module 2_3) was also conducted. Finally, the key rate‐limiting P450 monooxygenase from Module 1, which transforms CH to CHOL, was integrated to create the recombinant *E. coli* (Module 1_2_3), allowing for the direct production of AA from CH. During this process, extensive screening was carried out to identify the optimal P450 enzyme. Additionally, the intracellular supply of cofactor (NADPH/NADH) was enhanced by genetic modifications of *E. coli*. In the final stage, the reactions were scaled up and optimized to maximize AA production from either CHOL (or KA oil) or CH.

### 2.2 Construction of *E. coli* (Module 3) for Converting ε‐CL to AA

First, the *E. coli* (Module 3) was engineered to facilitate the biotransformation from ε‐CL to AA. This process involves the ring‐opening hydrolysis of ε‐CL to 6‐HHA catalyzed by lactonase from *Rhodococcus*,^[^ ^19^ ^]^ followed by two sequential oxidation steps to AA using ChnD and ChnE enzymes from *Acinetobacter*.^[^ ^20^, ^21^ ^]^ Our previous study indicated that these enzymes displayed higher catalytic activity compared to those in Modules 1 and 2, suggesting that lower expression levels might be adequate for the catalytic process. Thus, we opted for genome integration of genes encoding Lactonase, ChnD, and ChnE instead of using plasmids for enzyme expression, with the goal of alleviating expression burdens and facilitating compatibility to subsequent modules. Then, the selection of integration sites in the *E. coli* genome was guided by specific criteria: 1) ensuring no disruption of normal metabolic functions, 2) no impact on the AA production pathway, as well as 3) considering genes that could enhance the supply of intracellular cofactors (NAD^+^/NADP^+^). Consequently, we selected the NAD^+^‐dependent *ldhA* gene (encoding D‐lactate dehydrogenase) and the *adhE* gene (encoding aldehyde‐alcohol dehydrogenase),^[^ ^22^, ^23^ ^]^ NADP^+^‐dependent *ahr* gene (encoding aldehyde reductase),^[^ ^24^ ^]^ along with the *pgi* (encoding glucose‐6‐phosphate isomerase) as the integration sites.^[^ ^25^, ^26^ ^]^

**Figure 3:**
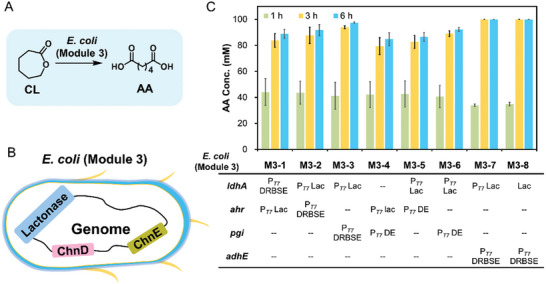
Biotransformation of ε‐CL to AA by engineered *E. coli* (Module 3) with enzyme genes integrated into the genome. A) Scheme of conversion of ε‐CL to AA. B) Construction of single‐cell *E. coli* with genome integration approach. C) Biotransformation of ε‐CL to AA by engineered *E. coli* (Module 3) cells: *E. coli* (M3‐1) ∼ *E. coli* (M3‐8). As shown in the table, *ldhA*, *ahr*, *pgi*, *adhE* was selected as the integration sites (replaced by exogenous genes). P*_T7_* DRBSE: Under the control of the P*_T7_* promoter, genes encoding ChnD and ChnE were linked by the RBS sequence; P*_T7_* Lac: Gene encoding Lactonase was under the control of P*_T7_* promoter; P*_T7_* DE: Proteins ChnD and ChnE were fused with linker, and the resulting ChnDE fusion gene was driven by P*_T7_* promoter; Lac: Gene encoding Lactonase was under the control of the native promoter of the gene that was deleted at the integration site in the engineered strain. Reactions were conducted at 25 °C and 220 rpm using *E. coli* cells (16 g CDW L^−1^) expressing target enzymes in 200 mm phosphate buffer (pH 8.0) with 100 mm ε‐CL. The error bars indicate the standard deviation of three biological replicates (*n* = 3).

To start with, the gene encoding lactonase was first integrated at the *ldhA* site, using either the P*_T7_* promoter or its native promoter. The biotransformation achieved a complete conversion of 100 mm ε‐CL to 6‐HHA, significantly outperforming the control *E. coli* BL21(DE3), which produced only 20 mm 6‐HHA due to the hydrolysis of ε‐CL catalyzed by endogenous hydrolases (Figure S1, Supporting Information). Following this, genes encoding ChnD, ChnE, and a fusion of ChnD and ChnE (linked by SGGSGGSGGSAG, named ChnDE) were further integrated into other sites such as *ahr, pgi* or *adhE*. Consequently, all engineered *E. coli* (Module 3) strains exhibited high catalytic efficiency in ε‐CL‐to‐AA conversion, producing ≥ 80 mm (12 g L^−1^) AA within 6 h (**Figure** **3**C). Among them, strains with the *pgi* gene knocked out, such as *E. coli* (M3‐3), *E. coli* (M3‐4), and *E. coli* (M3‐6), exhibited slower growth rates and lower biomass, likely due to disruption in glycolysis. In contrast, *E. coli* strains *E. coli* (M3‐7) and *E. coli* (M3‐8), which had the genes of lactonase, ChnD, and ChnE integrated at *ldhA* and *adhE* sites, achieved a complete substrate conversion, producing ca. 100 mm AA (14.6 g L^−1^). Although SDS‐PAGE analysis did not reveal distinct expression bands for lactonase, ChnD, ChnE, and ChnDE (Figure S2A, Supporting Information), subsequent transcriptional analysis showed a significant drop in transcript levels compared to those using plasmids for enzyme overexpression, yet still higher than the control *E. coli* BL21(DE3) (Figure S2B, Supporting Information). In summary, the integration of these genes into *the* genome resulted in low enzyme expression levels while maintaining high catalytic efficiency.

### 2.3 Construction of *E. coli* (Module 2_3) for Converting CHOL to AA

Subsequently, *E. coli* (Module 2_3) was developed by integrating Module 2 enzymes into Module 3 for CHOL‐to‐AA conversion, featuring ADH‐mediated CHOL‐to‐CHONE oxidation and BVMO‐catalyzed CHONE‐to‐ε‐CL transformation. Due to the less‐than‐ideal activity of these enzymes, a strategy to improve their expression was implemented.

**Figure 4:**
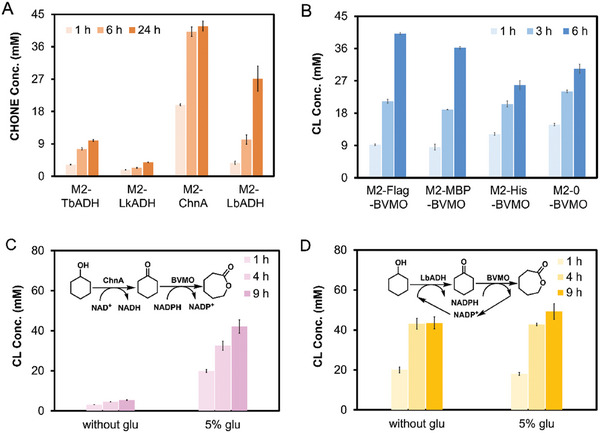
Construction of recombinant *E. coli* catalyst for converting CHOL to ε‐CL. A) *E. coli* strains expressing various ADHs were evaluated for CHOL‐to‐CHONE biotransformation under the following conditions: 25 °C, 220 rpm, 8 g CDW L^−1^ cell density in 200 mm phosphate buffer (pH 8.0) with 50 mm CHOL. B) *E. coli* strains expressing BVMO fused different tags were evaluated for CHONE‐to‐ε‐CL biotransformation under the following conditions: 25 °C, 220 rpm, 8 g CDW L^−1^ cell density in 200 mm phosphate buffer (pH 8.0) with 50 mm CHONE; cofactor NAD(P)H was provided by the *E. coli* using 5% (w/v) glucose as an energy source. C) *E. coli* (Module 2) co‐expressing ChnA and BVMO were evaluated for CHOL‐to‐ε‐CL biotransformation under the following conditions: 25 °C, 220 rpm, 8 g CDW L^−1^ cell density in 200 mm phosphate buffer (pH 8.0) with 50 mm CHOL, with or without the addition of 5% (w/v) glucose. D) *E. coli* (Module 2) co‐expressing LbADH and BVMO were evaluated for CHOL‐to‐ε‐CL biotransformation under the following conditions: 25 °C, 220 rpm, 8 g CDW L^−1^ cell density in 200 mm phosphate buffer (pH 8.0) with 50 mm CHOL, with or without the addition of 5% (w/v) glucose. The error bars indicate the standard deviation of three biological replicates (*n* = 3).

First, four alcohol dehydrogenase (ADH) candidates (LkADH,^[^ ^27^ ^]^ TbADH,^[^ ^28^ ^]^ ChnA,^[^ ^21^ ^]^ and LbADH)^[^ ^29^ ^]^ previously reported to be effective in CHOL oxidation were expressed in *E. coli* and evaluated for their capability to convert CHOL (50 mm) to CHNOE. As presented in **Figure** **4**A, ChnA, derived from the CHOL‐degrading strain *Acinetobacter* sp. Strain SE19^[^ ^21^ ^]^ emerged as the most active candidate, producing ca. 40 mm CHONE within 6 h. This superior catalytic performance is linked to ChnA's dependency on NAD^+^ that is more readily available in *E. coli* than NADP^+^, as well as its natural affinity for CHOL.^[^ ^27^, ^28^, ^29^, ^30^, ^31^, ^32^ ^]^ While among the other ADHs, LbADH from *Lactobacillus brevis* exhibited the highest catalytic efficiency. Subsequently, in the conversion of CHOL to CHONE, the BVMO variant (C376L/M400I), derived from CHMO from *Acinetobacter calcoaceticus*,^[^ ^33^ ^]^ showed a superior catalytic activity compared to *Tm*CHMO from *Thermocrispum municipal*,^[^ ^28^ ^]^ (Figure S3, Supporting Information). To further improve the stability and solubility of BVMO, a tag fusion strategy was implemented, which involved individually fusing maltose‐binding protein (MBP) with an attached E6,^[^ ^34^ ^]^ along with a flag‐tag or his‐tag, to the N‐terminal of BVMO. The results showed that the BVMO fused with the flag tag displayed the best catalytic activity with the highest protein expression level (Figure 4B; Figure S4, Supporting Information), yielding ca. 40 mm ε‐CL from 50 mM CHONE. Consequently, ChnA and LbADH, which have different cofactor preferences, were co‐expressed with flag‐tagged BVMO in *E. coli* BL21(DE3), respectively. The results indicated that the use of NAD^+^‐dependent ChnA (Figure 4C) resulted in ca. 5.4 mm ε‐CL after 9 h reaction, which was likely due to insufficient regeneration of endogenous NADPH to facilitate the BVMO‐catalyzed reaction.^[^ ^35^ ^]^ However, the addition of exogenous glucose could boost NADPH production through *E. coli*’s central metabolism, leading to ≈40 mm of ε‐CL. In contrast, a combination of LbADH with BVMO formed a redox‐neutral system via a hydrogen‐borrowing mechanism (Figure 4D), achieving an excellent conversion of CHOL to ε‐CL (42 mm) without the need for extra addition of cofactors and glucose. Furthermore, it was observed that glucose had minimal impact on the productivity of LbADH and BVMO catalyzed reactions, as similar yields were achieved in both cases (with or without glucose addition).

**Figure 5:**
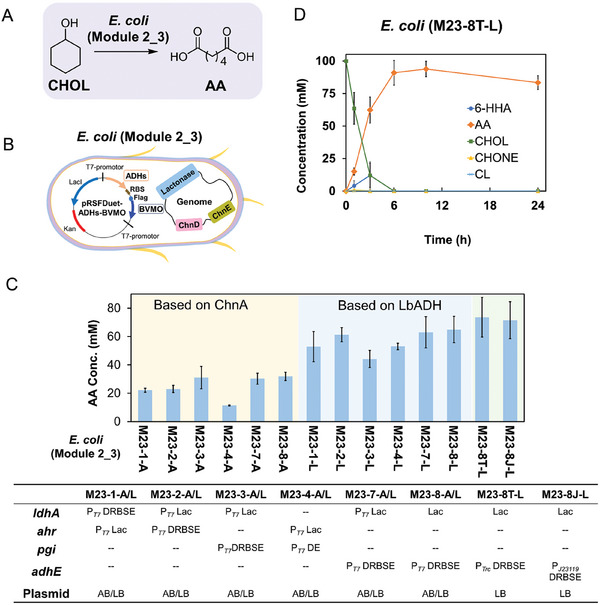
Biotransformation of CHOL to AA with engineered *E. coli* (Module 2_3). A) Scheme of biotransformation from CHOL to AA. B) Construction of single *E. coli* containing enzymes of Module 2_3 for converting CHOL to AA. C) Different engineered *E. coli* (Module 2_3) cells were evaluated for CHOL‐to‐AA biotransformation under the following conditions: 25 °C, 220 rpm, 16 g CDW L^−1^ cell density in 200 mm phosphate buffer (pH 8.0) with 150 mm CHOL for 24 h. AB: Plasmid pRSFDuet‐1 which carries both ChnA and flag‐tagged BVMO; LB: Plasmid pRSFDuet‐1 which carries both LbADH and flag‐tagged BVMO. For engineered *E. coli* based on ChnA (*E. coli* (M23‐1‐A); *E. coli* (M23‐2‐A); *E. coli* (M23‐3‐A); *E. coli* (M23‐4‐A); *E. coli* (M23‐7‐A) and *E. coli* (M23‐8‐A)), 5% (w/v) glucose was added as an energy source to provide NAD(P)H; for engineered *E. coli* based on LbADH (*E. coli* (M23‐1‐L); *E. coli* (M23‐2‐L); *E. coli* (M23‐3‐L); *E. coli* (M23‐4‐L); *E. coli* (M23‐7‐L) and *E. coli* (M23‐8‐L)), no glucose was added to the reaction systems. D) Time course of recombinant *E. coli* (M23‐8T‐L) catalyzed CHOL‐to‐AA biotransformation under the following conditions: 25 °C, 220 rpm, 16 g CDW L^−1^ cell density in 200 mm phosphate buffer (pH 8.0) with 100 mm CHOL. The pH was maintained ≈8.0 through the addition of 10 m NaOH. The error bars indicate the standard deviation of three biological replicates (*n* = 3).

Then, the plasmid pRSFDuet‐1, which harbors the optimal combination of ChnA or LbADH along with flag‐tagged BVMO (Figure S5, Supporting Information), was separately introduced into *E. coli* (Module 3) to generate the recombinant *E. coli* (Module 2_3) for converting CHOL to AA. The biotransformation without glucose addition showed that the combination of LbADH and BVMO generally exhibited better catalytic performance than those of ChnA and BVMO, likely due to a more effective cofactor regeneration (redox‐neutral system) formed between LbADH and BVMO, which eliminated the need for glucose supplementation. Among the engineered *E. coli* (Module 2_3) with LbADH and BVMO combinations, *E. coli* (M23‐8‐L) showed the highest catalytic activity, producing ca. 65 mm AA. To further improve the AA production and minimize the formation of intermediate product 6‐HHA, the ChnD‐catalyzed oxidation of 6‐HHA was further enhanced by testing different promoters of ChnD with variations of strengths and corresponding RBS. The results showed that *E. coli* (M23‐8T‐L) with the promoter changed from P*_T7_* to P*_trc,_* demonstrated the highest activity, converting 150 mm CHOL to ca. 73 mm AA (**Figure** **5**C). Thus, *E. coli* (M23‐8T‐L) was selected as a whole‐cell catalyst for subsequent reactions, successfully converting up to 100 mm CHOL to produce ca. 94 mm AA (94% yield) without the need for external cofactors or co‐substrates. In addition, no intermediates were detected during the reaction (Figure 5D).

### 2.4 Scale‐Up of the Bioprocess from CHOL or KA‐oil to AA

**Figure 6:**
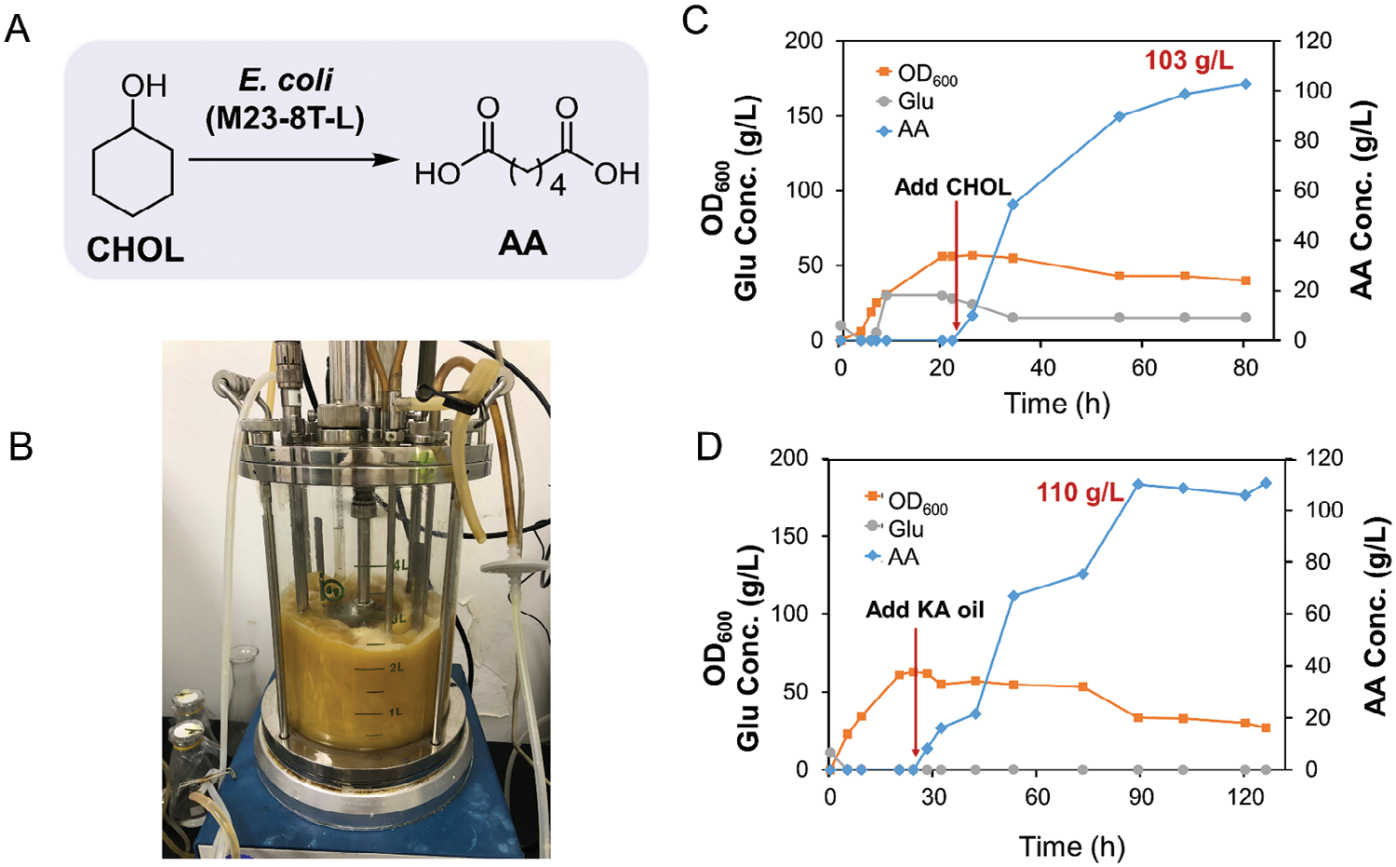
Biotransformation of CHOL to AA with engineered *E. coli* (M23‐8T‐L) in a 5‐L fermenter. A) Scheme of biotransformation from CHOL to AA by engineered *E. coli* (M23‐8T‐L); B) Biotransformation of CHOL into AA in a 5‐L fermenter; C) Biotransformation results for *E. coli* (M23‐8T‐L) catalyzed conversion of CHOL to AA. D) Biotransformation results for *E. coli* (M23‐8T‐L) catalyzed conversion of industrially produced KA oil into AA. Refer to the experimental section for detailed reaction conditions. Arrow: the addition of CHOL or KA oil for initiating the reactions.

With the optimized strain of *E. coli* (M23‐8T‐L) in hand, scale‐up reactions were conducted in a 5‐L bioreactor using either CHOL or KA oil as starting material. A two‐stage fermentation process was employed, consisting of a cultivation phase for cell growth and enzyme expression with glucose addition as an energy source, followed by a biotransformation phase with fed‐batch substrate addition. As illustrated in **Figure** **6**C,D, using CHOL as substrate, productivity of ca. 103 g L^−1^ AA was achieved after an 80 h reaction, with no intermediates detected. Similarly, when using industrially produced KA oil as substrate, the AA concentration reached ≈110 g L^−1^, also with no intermediate accumulation. Furthermore, after the reaction, AA was isolated through a series of processes, including centrifugation, flocculation, membrane ultrafiltration, and recrystallization following a pH adjustment to below 2. Finally, the purity of the isolated AA exceeded 98.6%, with a yield of over 75%. In summary, the engineered *E. coli* (M23‐8T‐L) effectively converted both CHOL and industrially produced KA oil into AA at the product titers above 100 g L^−1^, eliminating the need for nitric acid and significantly reducing greenhouse gas emissions. This achievement represents the highest reported titer for AA biosynthesis and offers considerable potential for industrial application, by providing an option that integrates both chemocatalysis and biocatalysis for AA production starting from CH (Figure 1B).

### 2.5 Construction of *E. coli* (Module 1_2_3) for Converting CH to AA

**Figure 7:**
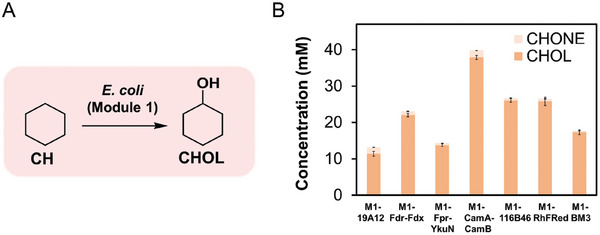
Biotransformation of CH to CHOL by P450CHX in the presence of different redox pairs or fused with reductase domain derived from self‐sufficient P450s. A) Scheme of conversion of CH to CHOL catalyzed by P450CHX. B) The hydroxylation of CH catalyzed by P450 CHX when coupled with different pairs of redox partners or fused with the reductase domain from different sources. Redox partners: Fdr‐Fdx derived from *Synechococcus elongatus* PCC7942;^[^ ^39^ ^]^ Fpr‐YkuN derived from *Bacillus subtilis*;^[^ ^40^ ^]^ CamA‐CamB derived from *Pseudomonas putida*.^[^ ^37^ ^]^ Reductase domains were from self‐sufficient P450s: 116B46 was reductase domain of CYP116B46 from thermophilic *Tepidiphilus thermophilus*;^[^ ^38^ ^]^ BM3 was reductase domain of CYP102A1 from *Bacillus megaterium*;^[^ ^18^ ^]^ RhFRed was reductase domain of P450RhF from *Rhodococcus* sp. strain NCIMB 9784;^[^ ^38^ ^]^ 19A12 was reductase domain of CYP102A1 mutant 19A12.^[^ ^18^ ^]^ *E. coli* co‐expressing P450CHX and different redox partners or expressing the chimeric P450CHX were evaluated for CH‐to‐CHOL biotransformation under the following conditions for 20 h: 25 °C, 220 rpm, 16 g CDW L^−1^ cell density in 200 mm phosphate buffer (pH 8.0) with 100 mm CH and 5% (w/v) glucose added. The error bars indicate the standard deviation of three biological replicates (*n* = 3).

Finally, to enable the conversion of CH to AA, the P450 monooxygenase of Module 1 was introduced into *E. coli* (Module 2_3) to generate the engineered *E. coli* (Module 1_2_3). The P450‐catalyzed hydroxylation of CH to CHOL, a key rate‐limiting step in the synthesis of AA from CH, was optimized using cytochrome P450CHX from *Acidovorax* sp. CHX100.^[^ ^36^ ^]^ It was known that most of the P450 monooxygenases must be associated with redox partners (ferredoxin and ferredoxin reductase) for transferring electrons from cofactors like NAD(P)H to the heme domain, a process critical for catalyzing the desired reactions and often serves as the rate‐limiting factor in the catalytic cycle. Here, as a three‐component P450 system, P450CHX activity was reconstituted by either adding different redox partners or fusing it to different reductase domains of reported self‐sufficient P450s (CYP116B46, CYP102A1 or P450RhF) to generate the chimeric enzyme with redox partners in a single polypeptide. As shown in **Figure** **7**B, the co‐expression of the reductase‐ferredoxin pair (CamA‐CamB)^[^ ^37^ ^]^ with P450CHX resulted in the highest catalytic activity, producing 40 mm CHOL from 50 mm CH. In contrast, the P450CHX chimeric enzymes P450CHX‐116B46 and P450CHX‐RhFRed^[^ ^38^ ^]^ displayed lower activity compared to the P450CHX and CamA‐CamB combination. Therefore, the Cam A and CamB redox pair was selected for use in the subsequent experiments.

**Figure 8:**
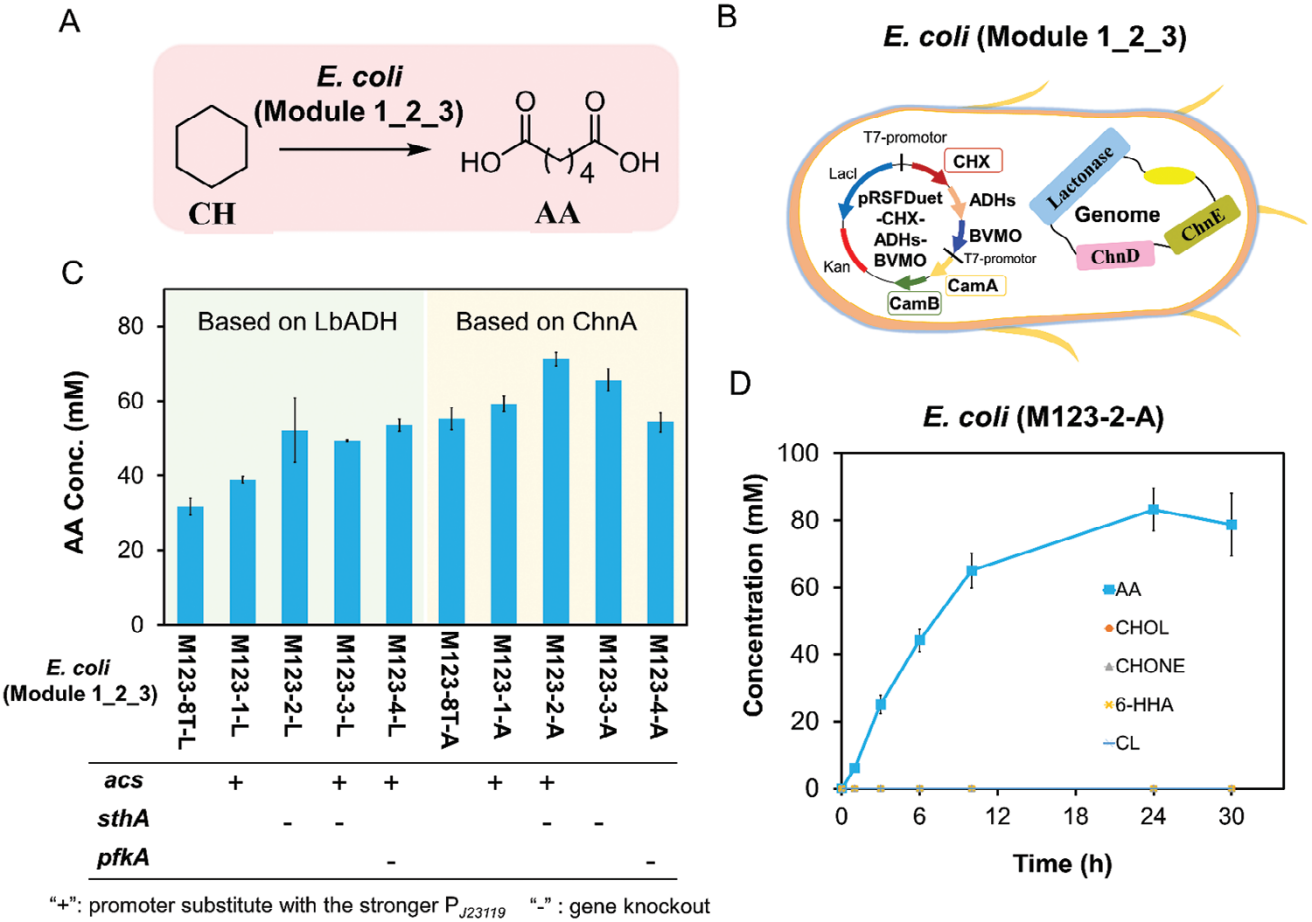
Biotransformation of CH to AA by engineered *E. coli* (Module1_2_3). A) Scheme of conversion from CH to AA. B) A single *E. coli* strain (Module1_2_3) was engineered to express all enzymes required for CH‐to‐AA conversion. C) Different engineered *E. coli* for biotransformation from CH by optimizing cofactor supply at the cellular level. D) Time course of recombinant *E. coli* (M123‐2‐A) catalyzed CH‐to‐AA biotransformation under the following conditions: 25 °C, 220 rpm, 16 g CDW L^−1^ cell density in 200 mm phosphate buffer (pH 8.0) with 100 mm CH and 5% (w/v) glucose added. The pH was maintained ≈8.0 through the addition of 10 m NaOH. The error bars indicate the standard deviation of three biological replicates (*n* ≥ 3).

Next, the genes encoding P450 CHX, Cam A, and CamB were further integrated into the plasmid pRSFDuet‐1 that already carried the enzyme genes of Module 2 (Figure S5, Supporting Information). The resulting two plasmids, differentiated by LbADH or ChnA, were then transformed into the *E. coli* (Module 3), yielding two recombinant *E. coli* (Module 1_2_3): *E. coli* (M123‐8T‐L) (containing NADP^+^ dependent LbADH) and *E. coli* (M123‐8T‐A) (expressing NAD^+^‐dependent ChnA) (**Figure** **8**B). With the addition of glucose as an energy source, the biotransformation results showed that *E. coli* (M123‐8T‐L) produced approximately 32 mm AA from 100 mm CH, while *E. coli* (M123‐8T‐A) yielded ≈55 mm AA.

To further improve the catalytic efficiency of engineered *E. coli* (Module 1_2_3), the cofactor supply was optimized at the cellular level. First, to minimize the inevitable accumulation of acetic acid during *E. coli* cultivation, the expression of Acs (acetyl‐CoA synthetase) in *E. coli* (M123‐8T‐L) was enhanced by replacing its promoter with the stronger P*_J23119_* promoter.^[^ ^41^, ^42^, ^43^ ^]^ Consequently new recombinant *E. coli* (M123‐1‐L) with reduced acetic acid accumulations was generated (Figure S6, Supporting Information). Subsequently, the same strategy was applied to *E. coli* (M123‐8T‐A), resulting in the development of a new strain *E. coli* (M123‐1‐A). The biotransformation results with *E. coli* (M123‐1‐L) and *E. coli* (M123‐1‐A) revealed a slight increase in AA production, reaching 39 and 59 mm, respectively. Next, the genes *sthA*, encoding transhydrogenase isomers responsible for interconversion between NADH and NADP,^[^ ^26^, ^44^, ^45^ ^]^ and *pfkA*, encoding 6‐phosphofructokinase in the glycolytic pathway,^[^ ^46^, ^47^, ^48^ ^]^ were individually knocked out, hoping to enhance the cofactor supply in *E. coli*. As illustrated in Figure 8C, the *pfkA*‐knockout strain *E. coli* (M123‐2‐L) achieved ca. 54 mm AA starting from 100 mm CH, while the *sthA*‐knockout strain *E. coli* (M123‐2‐A) yielded an AA concentration of ca. 71 mm. Thus, the time course of best *E. coli* (M123‐2‐A)‐catalyzed conversion of CH to AA was monitored with pH maintained at 8.0. As shown in Figure 8D, the production rate of AA was fast within the first 8 h, reaching a maximum of 83 mm AA at 24 h. This represents a significant improvement, being ca.20‐fold higher than that of the production from the initially constructed single *E. coli* (4 mm).^[^ ^18^ ^]^

### 2.6 Scale‐Up of the Bioprocess from CH to AA

**Figure 9:**
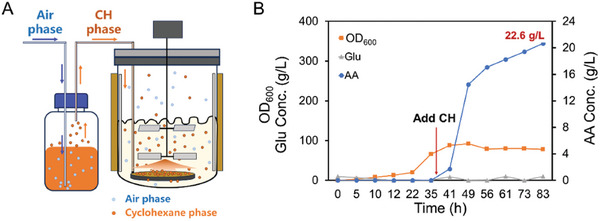
Biotransformation of CH to AA in a 5‐L fermenter with the best‐engineered *E. coli* (M123‐2‐A). A) Bioreactor setup for CH feed by the gas phase. B) Results of *E. coli* (M123‐2‐A) catalyzed biotransformation of CH to AA. Refer to the experimental section for detailed reaction conditions.

Ultimately, the production of AA from CH was carried out in a 5‐L bioreactor using the best‐engineered *E. coli* (M123‐2‐A). Throughout the culture process, parameters were controlled in line with those previously established for AA synthesis from CHOL. As shown in **Figure** **9**B, the growth rate of *E. coli* (M123‐2‐A) was slower than that of *E. coli* (M23‐8T‐L), with an OD_600_ reaching 20 only after 20 h. Subsequently, isopropyl β‐D‐thiogalactoside (IPTG) and 5‐aminolevulinic acid (5‐ALA) were added to a final concentration of 0.2 mm, followed by incubation at 25 °C for 18–20 h to induce protein expression, initiating the biotransformation process.

Given the high volatility and hydrophobic nature of CH, a gas dispersion method was employed to introduce CH, aiming to increase the residence time in the fermenter. Remarkably, the engineered *E. coli* (M123‐2‐A) continued to grow after the CH addition. During the initial 8 h, the product formation rate of AA reached ca. 1.31 g L^−1 ^h^−1^, which is comparable to the rate reported for the engineered *Pseudomonas taiwanensis* VLB120 (ca.1.28 g L^−1 ^h^−1^). However, after 83 h, the accumulation of AA reached ca. 22.6 g L^−1^ (155 mm), which was over 2‐fold higher than the titer achieved by *Pseudomonas taiwanensis* VLB120 (10.2 g L^−1^). Additionally, there are no detectable byproducts or intermediates formed during the biotransformation. To our knowledge, this study achieves the highest reported CH‐to‐AA bioconversion titers to date.

### 2.7 Substrate Scope Examination with Engineered *E. coli*

**Figure 10:**
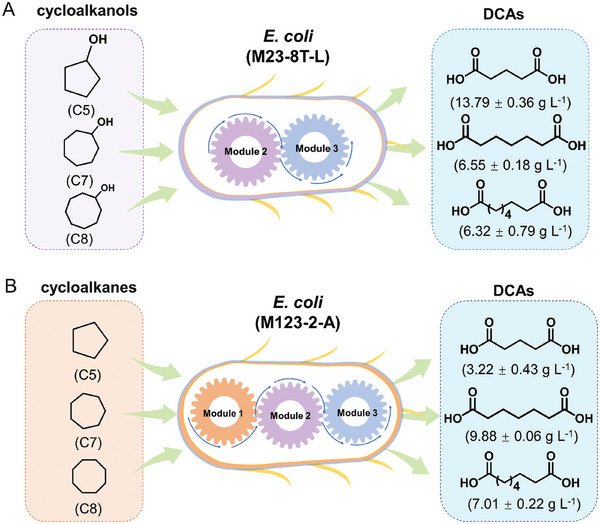
Substrate scope examination. A) The production of DCAs with engineered *E. coli* (M23‐8T‐L) from cycloalkanols. B) The production of DCAs with engineered *E. coli* (M123‐2‐A) from cycloalkanes. Reactions were performed in 3 mL of cell suspensions containing 100 mm substrates at 16 g CDW L^−1^, 25 °C, and 220 rpm for 24 h. For *E. coli* (M23‐8T‐L), no glucose was added to the reaction systems. For *E. coli* (M123‐2‐A), 5% (w/v) glucose was added as an energy source to provide NAD(P)H. The errors indicate the standard deviation of three biological replicates (*n* = 3).

Following the successful synthesis of AA, the substrate scope of the genetically engineered *E. coli* was then examined. The results indicated that cycloalkanes or cycloalkanols with varied carbon lengths (C5–C8) can be accepted as good substrates for the synthesis of corresponding dicarboxylic acids (DCAs). When using cycloalkanols as substrates, *E. coli* (M23‐8T‐L) effectively converted various substrates into corresponding DCAs including glutaric acid (ca. 14 g L^−1^), pimelic acid (ca. 7 g L^−1^), and suberic acid (ca. 6 g L^−1^) (see **Figure** **10**A). Notably, cyclopentanol was completely converted without any intermediate accumulation. In contrast, in the case of using cycloalkanes as substrates, *E. coli* (M123‐2‐A) converted various substrates into corresponding products such as glutaric acid (ca. 3 g L^−1^), pimelic acid (ca. 10 g L^−1^), and suberic acid (ca. 7 g L^−1^), all without the formation of byproducts or intermediates (Figure 10B).

## 3 Conclusion

In summary, we have developed an efficient biocatalytic system using a single *E. coli* catalyst to convert either CH or CHOL (or industrially produced KA oil) into AA. To tackle the challenges of creating a single cell capable of co‐expressing multiple enzymes, several strategies were employed, including enzyme optimization, genome editing, protein fusion, gene knockout, and promoter replacement. These efforts were aimed at balancing enzyme expression and fine‐tuning cofactor availability, thus achieving the most effective *E. coli* for AA biosynthesis to date.

Utilizing CHOL or KA oil, AA production exceeded 100 g L^−1^ with the engineered *E. coli* (M23‐8T‐L), representing the highest reported productivity to date. This system not only offers an optimal solution to HNO_3_‐dependent industrial processes, but also paves the way for designing a new chem‐enzymatic route that combines the chemical oxidation of CH to KA oil with subsequent biotransformation to AA. When CH, an inexpensive and readily available starting material, was employed, AA production of ca. 22.6 g L^−1^ was achieved, which is over two‐fold higher than that of the titer from the engineered *Pseudomonas taiwanensis* VLB120 (10.2 g L^−1^).^[^ ^17^ ^]^ Moreover, using KA oil as the starting material markedly boosts AA yield, enhancing its industrial viability at this stage. Nevertheless, it should be noted that CHOL and KA oil are produced industrially through the oxidation of CH, indicating that the synthesis of AA from CH is inherently more cost‐effective, assuming high productivity can be achieved.

Therefore, despite these achievements, there remains potential for further improving the efficiency of the developed catalytic system, particularly when using CH as the starting material due to the substrate's high hydrophobicity, volatility, and difficulty in the quantification of the substrate. The following aspects can be envisioned: 1) Employing directed evolution to enhance the activity/thermostability of key rate‐limiting enzymes, such as P450CHX, which has a spacious active site pocket with considerable potential for modification. 2) Exploring other cellular editing sites, such as *pntAB* and *ppnk*, or introducing exogenous enzymes to boost cofactor and energy supply.^[^ ^35^ ^]^ 3) Modifying *E. coli*’s cell membrane to improve the uptake capacity for the CH substrate,^[^ ^49^ ^]^ thereby improving the reaction rate. Moreover, recent advancements in artificial intelligence (AI) technology and genome‐scale engineering techniques have provided us with exciting opportunities for future developments. Finally, we anticipate the design of more efficient biocatalytic systems/bioreactors tailored to specific needs, thus offering ideal alternatives to current chemical industrial processes for the synthesis of more high‐value‐added or important products.

## 4 Experimental Section

### Chemicals and Reagents

Isopropyl β‐D‐1‐thiogalactopyranoside (IPTG), L‐Arabinose, 5‐Aminolevulinic acid hydrochloride (5‐ALA), Kanamycin, Spectinomycin were purchased from Sangong (Shanghai, China); Tryptone and yeast extract were purchased from OXOID (Shanghai, China); Primer STAR Max DNA Polymerase was purchased from Takara (Shanghai, China); T5 exonuclease and Buffer 4.0 were obtained from New England Biolabs (Beverley, MA, USA); Plasmid and DNA purification kits were purchased from TransGen Biotech (Beijing, China). Gene synthesis was performed by GeneCreate Biological Engineering (Wuhan, China) while oligonucleotide synthesis and DNA sequencing were conducted by Sangon (Shanghai, China). All other chemicals were of analytical grade and commercially available. The *E. coli* strains were grown on lysogeny broth (LB) or terrific broth (TB) containing antibiotics with appropriate concentration (50 µg/mL kanamycin or 100 µg/mL spectinomycin).

### Medium for Scale‐Up (pH 7.0)

A total of 11 g L^−1^ glucose, 20 g L^−1^ tryptone, 10 g L^−1^ yeast extract, 1 g L^−1^ Na_2_HPO_4_, 2.1 g L^−1^ citric acid monohydrate, 0.82 g L^−1^ MgSO_4_·7H_2_O, 2 g L^−1^ (NH_4_)_2_SO4, 4 g L^−1^ K_2_HPO_4_, 1 mL L g L^−1^ TE stock solution.

TE stock solution (pH 7.5): 0.3 g L^−1^ CuSO_4_ **·**5H_2_O, 1.7 g L^−1^ MnSO_4_·H_2_O, 0.3 g L^−1^ ZnSO_4_·7H_2_O, 2.8 g L^−1^ FeSO_4_·7H_2_O, 2 g L^−1^ Na_2_MoO_4_·2H_2_O, 1 g L^−1^ H_3_BO_3_, 2.45 g L^−1^ CoCl_2_·6H_2_O, 2.45 g L^−1^ CaCl_2_.

### Strains and Plasmids

*E. coli* DH5α cells are used as cloning strains for DNA manipulation while *E. coli* BL21 (DE3) cells were used as hosts for biotransformation. The genes encoding alcohol dehydrogenases (LbADH from *Lactobacillus brevis*, ChnA from *Acinetobacter*, ChnD from *Acinetobacter*), aldehyde dehydrogenase (ChnE from *Acinetobacter*), lactonase from *Rhodococcus* sp. HI‐31, double mutant (C376L/M400I) of Baeyer‐Villiger monooxygenase (BVMO from *Acinetobacter* sp. NCIMB9871) and p450CHX from *Acidovorax* sp. CHX100 was chemically synthesized and ligated to plasmid pRSFDuet‐1. Details of strains, plasmids, primers, and synthetic gene sequences used in this study were summarized in Figure S5 and Tables S1–S6 (Supporting Information).

### Recombinant *E. coli* Construction

Enzyme‐encoding genes and linear plasmid backbones were PCR‐amplified using primers with 15–20 bp homologous arms for subsequent recombination. Genes were assembled via overlap PCR and cloned into linearized vectors using T5 exonuclease to generate 15 bp sticky ends, enhancing recombination efficiency. Specifically, the 5 µL reaction mixture, containing linear vector, enzyme genes, Buffer 4.0, and T5 exonuclease, was incubated on an ice‐water mixture for 5 min before adding 50 µL of *E. coli* DH5α competent cell for transformation. The transformants were selected on antibiotic‐containing LB agar plates and verified by DNA sequencing. Verified plasmids were transformed into engineered *E. coli* BL21 (DE3) cells for protein expression and whole‐cell biocatalyst preparation.

### CRISPR‐Cas9‐Mediated Gene Editing in *E. coli* BL21 (DE3) Cells

The general procedure was performed as previously described by Jiang et al.^[^ ^50^ ^]^ utilizing a system comprising the host strain (*E. coli* BL21(DE3)), plasmids (pCasd and pTarget), and donor DNA. Briefly, pCas‐transformed cells were induced with 20 mm arabinose to express λ‐Red recombinase for homologous recombination enhancement. Competent cells were prepared and co‐electroporated with pTarget (containing sgRNA) and donor DNA. Following recovery at 30 °C, cells were plated on LB agar with kanamycin (50 µg mL^−1^) and spectinomycin (100 µg mL^−1^) and incubated at 30 °C. Positive transformants were verified by colony PCR and sequencing. Plasmid curing was achieved by sequential removal of pTarget (using 50 µg mL^−1^ kanamycin and 0.5 mm IPTG) and pCas (37 °C overnight incubation). The resulting engineered strain was used for subsequent experiments.

### Whole‐Cell Biocatalyst Preparation

Engineered *E. coli* cells were initially cultured in 3 mL LB medium with kanamycin (50 µg mL^−1^) at 37 °C and 220 rpm for 6 h. The precultures (1 mL) were transferred to 50 mL TB medium with appropriate antibiotics and cultured at 37 °C, 220 rpm for 2–3 h until OD_600_ = 0.6–0.8. Protein expression was induced with 0.2 mm IPTG at 25 °C for 14–16 h. Cells were harvested by centrifugation (3040 × g, 15 °C for 10 min), washed with 200 mm potassium phosphate buffer (pH 8.0), and used as whole‐cell biocatalysts.

### Whole‐Cell Biotransformation Procedure

Reactions were conducted in 3 mL cell suspensions (potassium phosphate buffer, pH 8.0) at 25 °C and 220 rpm in 100 mL screw‐capped shake flasks. Typically, 5% (w/v) glucose was added as a co‐substrate for NAD(P)H regeneration. Samples were collected at designated intervals and prepared for GC analysis.

### GC Sample Preparation

For AA and 6‐HHA analysis, 50 µL reaction samples were mixed with 450 µL water, 50 µL HCl (4 m), and 500 µL EtOAc, followed by vortexing and centrifuging (13680 × g, 1 min). The organic phase was dried over anhydrous Na_2_SO_4_ for derivatization. For CHOL, CHONE, and ε‐CL analysis, 50 µL samples were extracted with 450 µL water and 500 µL EtOAc containing 2 mm n‐decane (internal standard), followed by similar processing.

### Scale‐Up Biotransformation in 5‐L Fermenter

Biotransformation of CHOL or CH to AA was scaled up using engineered *E. coli* in 5‐L fermenters (Baoxing Biotech, Shanghai, China) through two stages: cultivation and biotransformation. During the cultivation stage, conditions were maintained at 37 °C, pH 7.0, 1 vvm aeration, 300–700 rpm agitation, and dissolved oxygen (DO) at 15%‐30%. At OD_600_ = 20‐25, 0.2 mm IPTG was added and the temperature was reduced to 25 °C for 12–20 h. For biotransformation, pH was maintained ≈7.5 by adding NH_3_·H_2_O. In the conversion from CHOL to AA, CHOL was added employing fed‐batch addition (1 vvm, 600 rpm) with glucose maintained below 5 g L^−1^. In the conversion from CH to AA, CH was added using gas‐phase feeding (0.25 vvm, 600 rpm) from the bottom of the fermenter with glucose (10 g L^−1^) added when pH exceeded 7.6. Samples were collected periodically and prepared for GC analysis.

### Derivatization

EtOAc extracts were centrifugated (13 680 × g, 10 min), and 300 µL supernatant was derivatized with N‐methyl‐N‐(trimethylsilyl) trifluoroacetamide/pyridine (1:2) at 65 °C for 1 h.

### Analytical Methods

CHOL, CHONE and ε‐CL were analyzed using SHIMADZU Nexis GC‐2030 with SH‐Rtx‐5 column (30 m × 0.25 mm, 0.25 µm), injector at 250 °C, detector at 280 °C, and temperature program: 50 to 120 °C at 5 °C min^−1^, then to 240 °C at 40 °C min^−1^, and held at 240 °C for 1 min. Derivatized products of AA and 6‐HHA were analyzed similarly after adding 90 µL EtOAc containing an internal standard (25 mm n‐decane). The temperature program was: 5 °C per min from 50 to 120, 40 °C per min to 240 °C, and held at 240 °C for 1 min. The GC‐MS analysis of derivatized products of DCAs was performed using the SHIMADZU GCMS‐QP2010 SE with Rtx‐5MS column (30 m × 0.25 mm, 0.25 µm) (Figure S7, Supporting Information). Acetic acid concentration was quantified by HPLC (Shimadzu LC‐20AD) with a Bio‐Rad Aminex HPX‐87H column (Bio‐Rad, Hercules, CA, U.S.A.) and a refractive index detector at 35 °C, using 5 mm H_2_SO_4_ solution was used as the mobile phase (0.5 mL min^−1^).

### Statistical Analysis

Statistical analysis was performed using Excel (Microsoft Office 2019) and Prism8.0 (GraphPad). Results presented are mean ± standard deviations (SD) from at least three independent determinations, in which the error bars represent the standard error of the mean (SEM).

## Conflict of Interest

The authors declare no conflict of interest.

## Author Contributions

F.W. and H.S. contributed equally to this work. A.L., F.W., and Q.L. performed project conception and experimental design. F.W., H.S., D.D., Y.W., and J.Z. performed the experimental operation and sample collection. F.W., H.S., J.Z., and Q.L. performed data analysis. F.W. and H.S. wrote the original draft. A.L., F.W., and Q.L. performed writing review and editing. All experiments were conducted under the supervision of A.L. All authors contributed to design development, data interpretation, and manuscript editing work.

## Supporting information



Supporting Information

## Data Availability

The data that support the findings of this study are available from the corresponding author upon reasonable request.
