# Supplementary material for: Multidimensional Engineering of Escherichia coli for Efficient Adipic Acid Synthesis From Cyclohexane
Source: Adv Sci (Weinh). 2025 Feb 17;12(14):2411938. doi: 10.1002/advs.202411938 (PMC11984861; doi:10.1002/advs.202411938)
Supplement: Supplementary file 1 — Supporting Information [file ADVS-12-2411938-s001.docx]

**Supporting Information**

**Multidimensional Engineering of *Escherichia coli* for Efficient Adipic Acid Synthesis from Cyclohexane**

Fei Wang^1,2†^, Huiqi Sun^1†^, Di Deng^1^, Yuanqing Wu^1^, Jing Zhao^1^, Qian Li^1*^, Aitao Li^1*^

^1^State Key Laboratory of Biocatalysis and Enzyme Engineering, Hubei Key Laboratory of Industrial Biotechnology, School of Life Sciences, Hubei University, Wuhan, 430062, P. R. China

^2^School of Synthetic Biology, Shanxi University, Taiyuan, 030031, P. R. China

Address correspondence to Aitao Li, aitaoli@hubu.edu.cn or Qian Li, qianli@hubu.edu.cn

[Supplementary Tables 3](#_Toc184372417)

[Table S1. List of the *E. coli* (Module 3) strains. 3](#_Toc184372418)

[Table S2. List of the *E. coli* (Module 2) strains 4](#_Toc184372419)

[Table S3. List of the *E. coli* (Module 1) strains 5](#_Toc184372420)

[Table S4. List of the *E. coli* (Module 2_3) strains 6](#_Toc184372421)

[Table S5. List of the *E. coli* (Module 1_2_3) strains 8](#_Toc184372422)

[Table S6. Oligonucleotide sequences 10](#_Toc184372423)

[Supplementary Figures 14](#_Toc184372424)

[Figure S1. Biotransformation of ε-CL to 6-HHA by *E. coli* cells. 14](#_Toc184372425)

[Figure S2. Protein expression analysis of *E. coli* (Module 3) containing Lactonase, ChnD and ChnE. 14](#_Toc184372426)

[Figure S3. Biotransformation of CHONE to ε-CL by *E. coli* cells expressing BVMO or TmCHMO. 15](#_Toc184372427)

[Figure S4. SDS-PAGE analysis of whole-cell proteins of BVMO fused different tags expressed in *E. coli*. 15](#_Toc184372428)

[Figure S5. Plasmid configuration of pRSFDuet-1 containing the enzyme genes in *E. coli* (Module 2_3) and *E. coli* (Module 1_2_3). 16](#_Toc184372429)

[Figure S6. Analysis of acetic acid. 16](#_Toc184372430)

[Figure S7. GC-MS analysis of α, ω-dicarboxylic acids derivatization. 17](#_Toc184372431)

[Supplementary Nucleotide Sequence Information 18](#_Toc184372432)

## Supplementary Tables

# Table S1. List of the *E. coli* (Module 3) strains.

| **Strains** | **Plasmid** | **Genotype** | **source** |
| --- | --- | --- | --- |
| M3-1 | *--* | Δ*ahr*::P*_T7_ lactonase*, Δ*ldhA*::P*_T7_ chnD* RBS *chnE* | This study |
| M3-2 | *--* | Δ*ldhA*::P*_T7_ lactonase*, Δ*ahr*::P*_T7_ chnD* RBS *chnE* | This study |
| M3-3 | *--* | ΔldhA::P*_T7_* *lactonase*, Δ*pgi*::P*_T7_* *chnD* RBS *chnE* | This study |
| M3-4 | *--* | Δ*ahr*::P*_T7_ lactonase*, Δ*ldhA*::P*_T7_ chnD-*linker*-chnE* | This study |
| M3-5 | *--* | Δ*ldhA*::P*_T7_ lactonase*, Δ*ahr*::P*_T7_ chnD-*linker*-chnE* | This study |
| M3-6 | *--* | Δ*ldhA*::P*_T7_* *lactonase*, Δ*pgi*::P*_T7_* *chnD*-linker-*chnE* | This study |
| M3-7 | *--* | Δ*ldhA*::P*_T7_* *lactonase*, Δ*adhE*::P*_T7_* *chnD* RBS *chnE* | This study |
| M3-8 | *--* | Δ*ldhA*::*lactonase*, Δ*ahdE*::P*_T7_* *chnD* RBS *chnE* | This study |
| M3-8T | *--* | Δ*ldhA*::*lactonase,* Δ*ahdE*::P*_trc_ chnD* RBS *chnE* | This study |
| M3-8J | *--* | Δ*ldhA*::*lactonase,* Δ*ahdE*::P*_J23119_ chnD* RBS *chnE* | This study |
| M3-Lac1 | *--* | Δ*ldhA*::*lactonase* | This study |
| M3-Lac2 | *--* | Δ*ldhA*::P*_T7_ lactonase* | This study |

# Table S2. List of the *E. coli* (Module 2) strains

| **Strains** | **Plasmids** | **Genotype** | **source** |
| --- | --- | --- | --- |
| M2-LbADH | pRSFDuet-1 carrying LbADH  （pRSFDuet-1-LbADH） | *E. coli* BL21(DE3) | This study |
| M2-TbADH | pRSFDuet-1 carrying TbADH （pRSFDuet-1-TbADH） | *E. coli* BL21(DE3) | This study |
| M2-LkADH | pACYCDuet-1 carrying LkADH  (pACYCDuet-1-LkADH) | *E. coli* BL21(DE3) | This study |
| M2-ChnA | pRSFDuet-1 carrying ChnA  (pRSFDuet-1-ChnA) | *E. coli* BL21(DE3) | This study |
| M2-BVMO | pET-22b carrying BVMO  (pET-22b-BVMO) | *E. coli* BL21(DE3) | This study |
| M2-TmCHMO | pRSFDuet-1 carrying TmCHMO  (pRSFDuet-1-TmCHMO) | *E. coli* BL21(DE3) | This study |
| M2-0-BVMO | pRSFDuet-1 carrying BVMO  (pRSFDuet-1-BVMO) | *E. coli* BL21(DE3) | This study |
| M2-His-BVMO | pRSFDuet-1 carrying 6×His-tagged BVMO  (pRSFDuet-1-6×His/BVMO) | *E. coli* BL21(DE3) | This study |
| M2-Flag-BVMO | pRSFDuet-1 carrying Flag-tagged BVMO  (pRSFDuet-1-Flag/BVMO) | *E. coli* BL21(DE3) | This study |
| M2-MBP-BVMO | pRSFDuet-1 carrying MBP-tagged BVMO linked with E6  (pRSFDuet-1-MBP/E6/BVMO) | *E. coli* BL21(DE3) | This study |

# Table S3. List of the *E. coli* (Module 1) strains

| **Strains** | **Plasmids** | **Genotype** | **source** |
| --- | --- | --- | --- |
| M1-19A12 | pRSFDuet-1 carrying P450 CHX fused with reductase domain of P450BM3 19A12  (pRSFDuet-1-P450CHX/BM3 19A12) | *E. coli* BL21(DE3) | This study |
| M1-Fdr-Fdx | pRSFDuet-1 carrying P450 CHX and redox partners of Fdr and Fdx  (pRSFDuet-1-P450CHX-Fdr-Fdx) | *E. coli* BL21(DE3) | This study |
| M1-Fpr-YkuN | pRSFDuet-1 carrying P450 CHX and redox partners of Fpr and YkuN  (pRSFDuet-1-P450CHX-Fpr-YkuN) | *E. coli* BL21(DE3) | This study |
| M1-CamA-CamB | pRSFDuet-1 carrying P450 CHX and redox partners of CamA and CamB  (pRSFDuet-1-P450CHX-CamA-CamB) | *E. coli* BL21(DE3) | This study |
| M1-116B46 | pRSFDuet-1 carrying P450 CHX fused with reductase domain of CYP116B46  (pRSFDuet-1-P450CHX/116B46) | *E. coli* BL21(DE3) | This study |
| M1-RhFRed | pRSFDuet-1 carrying P450 CHX and reductase domain of P450RhFRed  (pRSFDuet-1-P450CHX/RhFRed) | *E. coli* BL21(DE3) | This study |
| M1-BM3 | pRSFDuet-1 carrying P450 CHX and reductase domain of P450BM3  (pRSFDuet-1-P450CHX/BM3) | *E. coli* BL21(DE3) | This study |

# Table S4. List of the *E. coli* (Module 2_3) strains

| **Strains** | **Plasmids** | **Genotype** | **source** |
| --- | --- | --- | --- |
| M23-1-A | pRSFDuet-1 carrying ChnA and Flag-tagged BVMO  (pRSFDuet-1-ChnA-Flag/BVMO) | Δ*ahr*::P*_T7_ lactonase*, Δ*ldhA*::P*_T7_ chnD* RBS *chnE* | This study |
| M23-2-A | pRSFDuet-1 carrying ChnA and Flag-tagged BVMO  (pRSFDuet-1-ChnA-Flag/BVMO) | Δ*ldhA*::P*_T7_ lactonase*, Δ*ahr*::P*_T7_ chnD* RBS *chnE* | This study |
| M23-3-A | pRSFDuet-1 carrying ChnA and Flag-tagged BVMO  (pRSFDuet-1-ChnA-Flag/BVMO) | Δ*ldhA*::P*_T7_* *lactonase*, Δ*pgi*::P*_T7_* *chnD* RBS *chnE* | This study |
| M23-4-A | pRSFDuet-1 carrying ChnA and Flag-tagged BVMO  (pRSFDuet-1-ChnA-Flag/BVMO) | Δ*ahr*::P*_T7_ lactonase*, Δ*ldhA*::P*_T7_ chnD-*linker*-chnE* | This study |
| M23-7-A | pRSFDuet-1 carrying ChnA and Flag-tagged BVMO  (pRSFDuet-1-ChnA-Flag/BVMO) | Δ*ldhA*::P*_T7_* *lactonase*,  Δ*adhE*::P*_T7_* *chnD* RBS *chnE* | This study |
| M23-8-A | pRSFDuet-1 carrying ChnA and Flag-tagged BVMO  (pRSFDuet-1-ChnA-Flag/BVMO) | Δ*ldhA*::*lactonase*, Δ*ahdE*::P*_T7_* *chnD* RBS *chnE* | This study |
| M23-1-L | pRSFDuet-1 carrying LbADH and Flag-tagged BVMO  (pRSFDuet-1-LbADH-Flag/BVMO) | Δ*ahr*::P*_T7_ lactonase*, Δ*ldhA*::P*_T7_ chnD* RBS *chnE* | This study |
| M23-2-L | pRSFDuet-1 carrying LbADH and Flag-tagged BVMO  (pRSFDuet-1-LbADH-Flag/BVMO) | Δ*ldhA*::P*_T7_ lactonase*, Δ*ahr*::P*_T7_ chnD* RBS *chnE* | This study |
| M23-3-L | pRSFDuet-1 carrying LbADH and Flag-tagged BVMO  (pRSFDuet-1-LbADH-Flag/BVMO) | Δ*ldhA*::P*_T7_* *lactonase*, Δ*pgi*::P*_T7_* *chnD* RBS *chnE* | This study |
| M23-4-L | pRSFDuet-1 carrying LbADH and Flag-tagged BVMO  (pRSFDuet-1-LbADH-Flag/BVMO) | Δ*ahr*::P*_T7_ lactonase*, Δ*ldhA*::P*_T7_ chnD-*linker*-chnE* | This study |
| M23-7-L | pRSFDuet-1 carrying LbADH and Flag-tagged BVMO  (pRSFDuet-1-LbADH-Flag/BVMO) | Δ*ldhA*::P*_T7_* *lactonase*,  Δ*adhE*::P*_T7_* *chnD* RBS *chnE* | This study |
| M23-8-L | pRSFDuet-1 carrying LbADH and Flag-tagged BVMO  (pRSFDuet-1-LbADH-Flag/BVMO) | Δ*ldhA*::*lactonase*, Δ*ahdE*::P*_T7_* *chnD* RBS *chnE* | This study |
| M23-8T-L | pRSFDuet-1 carrying LbADH and Flag-tagged BVMO  (pRSFDuet-1-LbADH-Flag/BVMO) | Δ*ldhA*::*lactonase,* Δ*ahdE*::P*_trc_ chnD* RBS *chnE* | This study |
| M23-8J-L | pRSFDuet-1 carrying LbADH and Flag-tagged BVMO  (pRSFDuet-1-LbADH-Flag/BVMO) | Δ*ldhA*::*lactonase,*  Δ*ahdE*::P*_J23119_ chnD* RBS *chnE* | This study |

# Table S5. List of the *E. coli* (Module 1_2_3) strains

| **Strains** | **Plasmids** | **Genotype** | **source** |
| --- | --- | --- | --- |
| M123-8T-L | pRSFDuet-1 carrying P450CHX, LbADH, Flag-tagged BVMO, CamA and CamB.  (pRSFDuet-1-P450CHX-LbADH-Flag/BVMO-CamA-CamB) | Δ*ldhA*::*lactonase,*  Δ*ahdE*::P*_trc_ chnD RBS chnE* | This study |
| M123-1-L | pRSFDuet-1 carrying P450CHX, LbADH, Flag-tagged BVMO, CamA and CamB.  (pRSFDuet-1-P450CHX-LbADH-Flag/BVMO-CamA-CamB) | Δ*ldhA*::*lactonase*,  Δ*ahdE*::P*_trc_* *chnD* RBS *chnE*, P*_J23119_* acs | This study |
| M123-2-L | pRSFDuet-1 carrying P450CHX, LbADH, Flag-tagged BVMO, CamA and CamB.  (pRSFDuet-1-P450CHX-LbADH-Flag/BVMO-CamA-CamB) | Δ*ldhA*::*lactonase*,  Δ*ahdE*::P*_trc_* *chnD* RBS *chnE*, Δ*sthA* | This study |
| M123-3-L | pRSFDuet-1 carrying P450CHX, LbADH, Flag-tagged BVMO, CamA and CamB.  (pRSFDuet-1-P450CHX-LbADH-Flag/BVMO-CamA-CamB) | Δ*ldhA*::*lactonase*,  Δ*ahdE*::P*_trc_* *chnD* RBS *chnE*, Δ*sthA*, P*_J23119_* *acs* | This study |
| M123-4-L | pRSFDuet-1 carrying P450CHX, LbADH, Flag-tagged BVMO, CamA and CamB.  (pRSFDuet-1-P450CHX-LbADH-Flag/BVMO-CamA-CamB) | Δ*ldhA*::*lactonase*,  Δ*ahdE*::P*_trc_* *chnD* RBS *chnE*, Δ*pfkA*, P*_J23119_* *acs* | This study |
| M123-8T-A | pRSFDuet-1 carrying P450CHX, ChnA, Flag-tagged BVMO, CamA and CamB.  (pRSFDuet-1-P450CHX-ChnA-Flag/BVMO-CamA-CamB) | Δ*ldhA*::*lactonase,*  Δ*ahdE*::P*_trc_ chnD RBS chnE* | This study |
| M123-1-A | pRSFDuet-1 carrying P450CHX, ChnA, Flag-tagged BVMO, CamA and CamB.  (pRSFDuet-1-P450CHX-ChnA-Flag/BVMO-CamA-CamB) | Δ*ldhA*::*lactonase*,  Δ*ahdE*::P*_trc_* *chnD* RBS *chnE*, P*_J23119_* acs | This study |
| M123-2-A | pRSFDuet-1 carrying P450CHX, ChnA, Flag-tagged BVMO, CamA and CamB.  (pRSFDuet-1-P450CHX-ChnA-Flag/BVMO-CamA-CamB) | Δ*ldhA*::*lactonase*,  Δ*ahdE*::P*_trc_* *chnD* RBS *chnE*, Δ*sthA*, P*_J23119_* *acs* | This study |
| M123-3-A | pRSFDuet-1 carrying P450CHX, ChnA, Flag-tagged BVMO, CamA and CamB.  (pRSFDuet-1-P450CHX-ChnA-Flag/BVMO-CamA-CamB) | Δ*ldhA*::*lactonase*,  Δ*ahdE*::P*_trc_* *chnD* RBS *chnE*, Δ*sthA* | This study |
| M123-4-A | pRSFDuet-1 carrying P450CHX, ChnA, Flag-tagged BVMO, CamA and CamB.  (pRSFDuet-1-P450CHX-ChnA-Flag/BVMO-CamA-CamB) | Δ*ldhA*::*lactonase*,  Δ*ahdE*::P*_trc_* *chnD* RBS *chnE*, Δ*pfkA* | This study |

# Table S6. Oligonucleotide sequences

| **Name** | **Sequence (5’ →3’)** |
| --- | --- |
| 16sRNA-F | CAAAGAGGGGGACCTTCGGG |
| 16sRNA-R | CATGGCTGCATCAGGCTTGC |
| QChnD-F | GTGGTGGTAAACGTCTGAGCCTG |
| QChnD-R | CACAACCAATCCACGGATGAACCAG |
| 6His-BVMO-F | CACCATCATCACCACATGtcacaaaaaatggattttgatgctatcgtgattg |
| BVMO-R | ATTCGGATCCTGGCttaggcattggcaggttgcttg |
| MBP-QL-F | ATATACCATGAAAATCGAAGAAGGTAAACTGGTAATCTGG |
| MBP-QL-R | CTTCGATTTTCATGGTATATCTCCTTATTAAAGTTAAACAAAATTATTTCTACAGGG |
| E6-BVMO-F | gaagaagaggaagaggagtcacaaaaaatggattttgatgctatcgtgat |
| MBP-E6-R | ctcctcttcctcttcttcAGTCTGCGCGTCTTTCAGGG |
| Flag-QL-F | GGAGATATACCATGgattataaagatgatgatgataaatcacaaaaaatggattttg |
| Flag-QL-R | aatcCATGGTATATCTCCTTATTAAAGTTAAACAAAATTATTTCTACAGGG |
| LBADH-F | CACCATCATCACCACATGAGCAATCGTCTGGATGGTAAAGTTG |
| LBADH-RBS-R | GATATATctccttAGGTACCTTACTGTGCGGTATAACCACCATCCAC |
| RBS-MBP-F | CTaaggagATATATCATGAAAATCGAAGAAGGTAAACTGGTAATCTGG |
| pRSF MCS2-F | GGTACCCTCGAGTCTGGTAAAGAAAC |
| pRSF MCS2-R | CATATGTATATCTCCTTCTTATACTTAACTAATATACTAAGATGGGG |
| MSC2-Fdr-F | TATAAGAAGGAGATATACATATGTTGAATGCGAGTGTGGC |
| Fdx-MCS2-R | TTACCAGACTCGAGGGTACCCTAGTAGAGGTCTTCTTCTTTGTGGGTT |
| MCS2-Fpr-F | GAAGGAGATATACATATGGCTGATTGGGTAACAGGCAAAG |
| YKuN-MCS2-R | AGACTCGAGGGTACCTTATGAAACATGGATTTTTTCCTTGTTCATATAATCTGCC |
| MCS2-CamA-F | GAAGGAGATATACATATGGTTAACGCCAATGATAATGTTGTTATCGTTGG |
| CamA RBS-R | GATATATCTCCTTAGGTACCTTAGGCGCTACTCAGTTCGG |
| RBS-CamB-F | GGTACCTAAGGAGATATATCATGAGCAAAGTGGTTTATGTTAGCCATG |
| CamB-MCS2-R | TACCAGACTCGAGGGTACCTTACCACTGACGATCCGGCAC |
| CHX Qtaa-F | GCGGCCGCATAATGCTTAAGTC |
| CHX Qtaa-R | GGCATTAATGCGAACCGGCAG |
| CHX 116B46-F | GTTCGCATTAATGCCCTGCGTCAGCCGGTGCG |
| 116B46-MCS1-R | GCATTATGCGGCCGCTTACAGGTCCAGAACCAGACGTTCGGTTTTAG |
| CHX-RhFRED-F | GTTCGCATTAATGCCcggcatcaaccggtcaccatc |
| RhFRED MCS1-R | AGCATTATGCGGCCGCtcagagtcgcagggccagc |
| CHX-19A12-F | GTTCGCATTAATGCCGgtCAGTCTGCTAAAAAAGTACGCAAAAAGGC |
| 19A12-MCS1-R | GCATTATGCGGCCGCTTACCCAGCCCACACGTCTTTTG |
| CHX-RBS-R | GATATATctccttAGGTACCTTAGGCATTAATGCGAACCGGCAG |
| BVMO-CHXQtaa-R | CTTAAGCATTATGCGGCCGCttaggcattggcaggttgcttgatatc |
| RBS-LBADH-F | GGTACCTaaggagATATATCATGAGCAATCGTCTGGATGGTAAAGTTG |
| RBS-ChnA-F | GGTACCTaaggagATATATCatgGAAAAGATCATGAGCAACAAATTCAAC |
| ChnA-RBS-R | GATATATctccttAGGTACCTTATTTGCTGGTATATGCACCATCAACAACATAC |
| acs A-F | GAGAAGCCGCCGAAAATCACCG |
| acs A-R | AAAGAGGAGAAATTAACTATGAGCCAAATTCACAAACACACC |
| acs B-F | AGTTAATTTCTCCTCTTTTTCGTGCTAGCATTATACCTAGGACTGAGCTAGCTGTCAACGTTAAATGTAGGGGTATTGGCAG |
| acs B-R | GCTTTCCAGGAGAGATATTGATCGGG |
| T7-Lactonase-F | CGGGATCTCGACGCTCTCCC |
| Lactonase-T7ter-R | CCTGAGGTTTCAGCAAAAAACCCCTCAAG |
| T7-ldhA-B-F | TTTTTTGCTGAAACCTCAGGAAGACTTTCTCCAGTGATGTTGAATCACATTTAAGC |
| ldhA-B-R | CAAGCAGAATCAAGTTCTACCATGCCGA |
| ldhA-A-F | TATAAGTTAATGTCTGTTTCGCGGTCGCC |
| ldhA-A-T7-R | GGGAGAGCGTCGAGATCCCGTCTTGCCGCTCCCCTGCAACC |
| ldhA-A-R | ATCAGAAAGCCCTGGAATAATCTTGCCGCTCCCCTGCAAC |
| Lactonase-R | TTATTCCAGGGCTTTCTGATACCATGCTGC |
| Lactonase-F | ATGACCAATATTAGCGAAACCCTGAGCAC |
| ldhA-B-F | GTTTCGCTAATATTGGTCATAAGACTTTCTCCAGTGATGTTGAATCACATTTAAGC |
| ahr-A-T7-F | GCCGCGTTATTATCTGCTAAATTTATTGATGGTG |
| ahr-A-T7-R | GGGAGAGCGTCGAGATCCCGAAATCATTCGCAGCGCTGATCT |
| ahr-B-T7-F | TTTTTTGCTGAAACCTCAGGTTTTTGGTCCTTCTCTGGTGTTGTTTG |
| ahr-B-T7-R | GGCTGCTCTGACTTTGATATCTGTTAATGC |
| petT7-ahr-B-F | GAAAGGAGGAACTATATCCGTTTTTGGTCCTTCTCTGGTGTTGTTTGG |
| petT7-ldhA-B-F | GAAAGGAGGAACTATATCCGAAGACTTTCTCCAGTGATGTTGAATCACATTTAAGC |
| petT7ter-R | CGGATATAGTTCCTCCTTTCAGCAAAAAACCC |
| petT7-pgi-B-F | GAAAGGAGGAACTATATCCGTCATCGTCGATATGTAGGCCGG |
| pgi-B-R | ACAATTTCCCTTCATTGAATGAATGGAGATTTACCCA |
| pgi-A-F | TTTTCAGCCTTGGCACAAGGGAAG |
| pgi-A-T7-R | GGGAGAGCGTCGAGATCCCGTGGTTTTAGTGCCGTTAGCGTAATGTTG |
| adhE-A-F | CTGTTTTTGTGGCCGTAAAGCAAGC |
| AdhE-T7-A-R | GGGAGAGCGTCGAGATCCCGGAAAAAATCCGCTTAATCAGTAGCGCTG |
| adhE-T7ter-B-F | GAAAGGAGGAACTATATCCGTGAATGCAGTCTGCTTGGTCGG |
| adhE-B-R | CACCACCACTTCTGGTACAGGTTCTG |
| J23119-ChnD-F | TTGACAGCTAGCTCAGTCCTAGGTATAATGCTAGCACGAAAAAGAGGAGAAATTAACATATGGGCAGCAGCCATCACCATC |
| J23119-A-R2 | AGGACTGAGCTAGCTGTCAAGGCAATATAAACGGCCCCTTCTG |
| Trc-ChnD-F | TTGACAATTAATCATCCGGCTCGTATAATGTGTGGAATTGTGAGATGAGAAAGAGGAGAAATTAACATATGGGCAGCAGCCATCACCATC |
| Trc-A-R2 | TCCACACATTATACGAGCCGGATGATTAATTGTCAAGGCAATATAAACGGCCCCTTCTG |
| gRNA-ldhA | CGAGTCCTTTGGCTTTGAGC |
| gRNA-ahr | GAAGTTTATGAGTACGATCC |
| gRNA-pgi | GGTGAGTGGAAAGGTTATAC |
| gRNA-adhE | AGCAGACTTCCTGGCGAACG |
| gRNA-acs | CATATTATTAACATCCTACA |

## Supplementary Figures


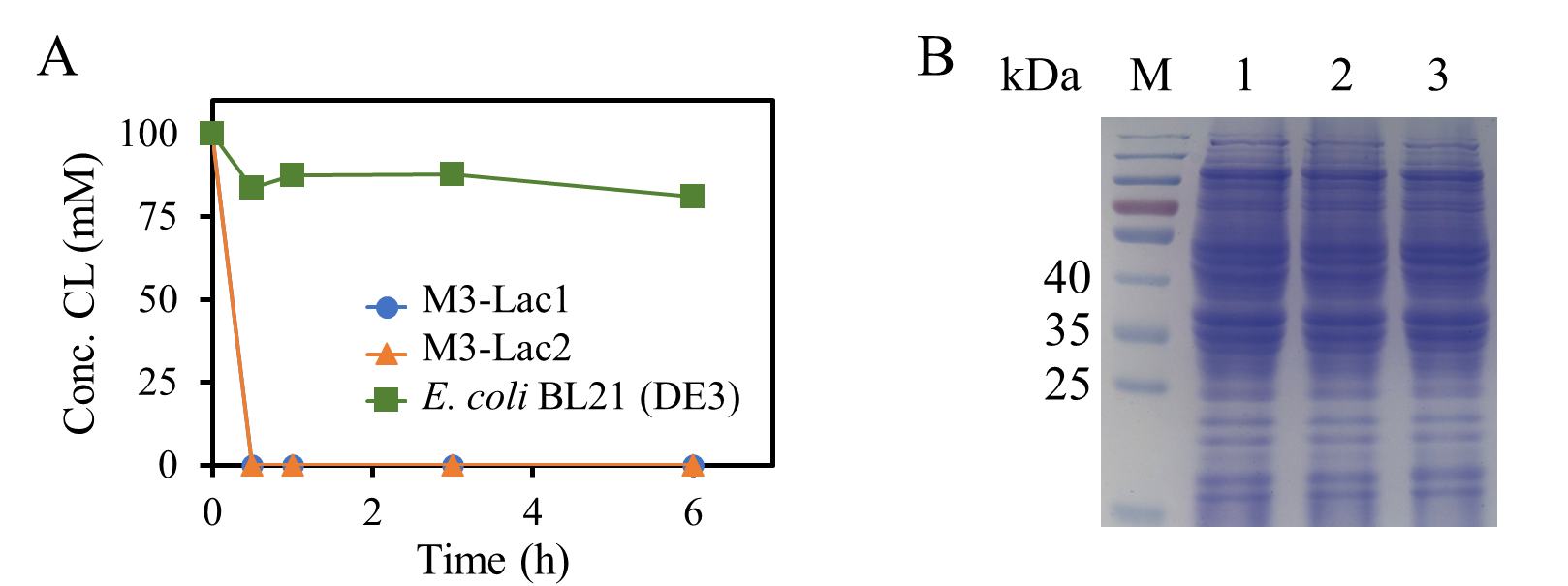


Figure S1. Biotransformation of ε-CL to 6-HHA by *E. coli* cells. **(A)** *E. coli*-catalyzed conversion of 100 mM CL to 6-HHA. M3-Lac1: Δ*ldhA*::*lactonase*; M3-Lac2: Δ*ldhA*::P*_T7_* *lactonase*. **(B)** SDS-PAGE analysis of whole-cell proteins in *E. coli*. Lane M: protein marker (Thermo Scientific 26616); Lane 1: M3-Lac1; Lane 2: M3-Lac2; Lane 3: *E. coli* BL21 (DE3). Lactonase: 33 kDa.

Figure S2. Protein expression analysis of *E. coli* (Module 3) containing **Lactonase, ChnD and ChnE***.* **(A)** SDS-PAGE analysis of whole-cell proteins of module 3 expressed in *E. coli*. Lactonase: 33 kDa, ChnD: 37 kDa, ChnE: 52 kDa. Lane M: protein marker (Thermo Scientific 26616); Lane 1:M3-DE (*E. coli* BL21 carrying plasmid pRSFDuet-1 which harbors ChnD and ChnE.); Lane 2: M3-1; Lane 3: M3-2; Lane 4: M3-3; Lane 5: M3-4; Lane 6: M3-5; Lane 7: M3-6; Lane 8: M3-7; Lane 9: M3-8. **(B)** RT-qPCR analysis for transcript levels of ChnD in different *E. coli* (Module 3) cells. 1: M3-DE; 2: M3-1;3: M3-2; 4: M3-3; 5: M3-4; 6: M3-5; 7: M3-6; 8: M3-7; 9: M3-8; 0: *E. coli* BL21(DE3). The 16S rRNA served as an internal control. Average values are from triplicate experiments.

Figure S3. Biotransformation of CHONE to ε-CL by *E. coli* cells expressing BVMO or TmCHMO. Reaction conditions: *E. coli* cells expressing BVMO or TmCHMO were resuspended in phosphate buffer (pH 8.0, 100 mM) at a cell density of 8 g CDW L^-1^, 50 mM CHONE, reactions were performed at 25°C, 220 rpm; cofactor NAD(P)H was provided by the *E. coli* cells using 5% glucose as an energy source. Average values are from triplicate experiments.


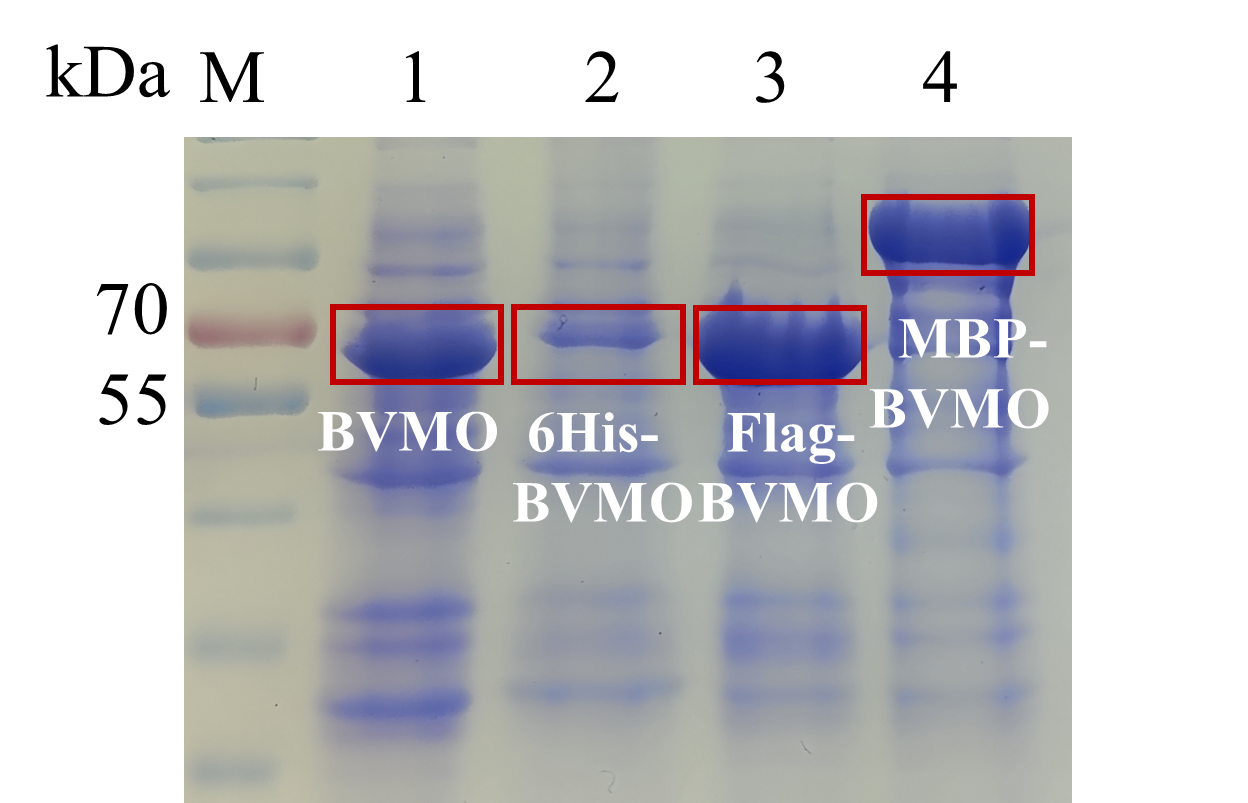


Figure S4. SDS-PAGE analysis of whole-cell proteins of BVMO fused different tags expressed in *E. coli*. Lane M: protein marker (Thermo Scientific 26616); Lane 1: M2-0-BVMO; Lane 2: M2-6His-BVMO; Lane 3: M2-Flag-BVMO; Lane 4: M2-MBP-BVMO.


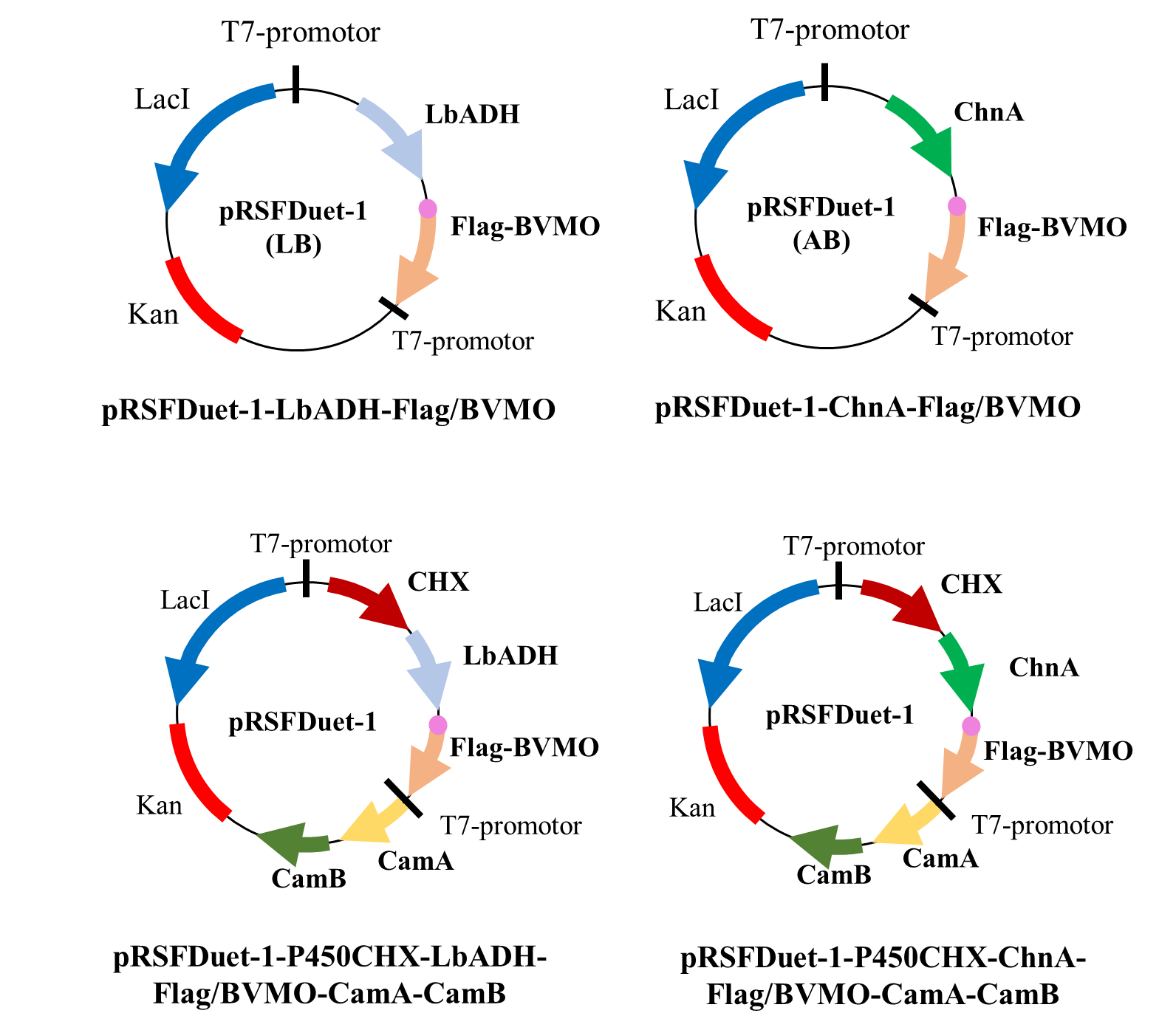


# Figure S5. Plasmid configuration of pRSFDuet-1 containing the enzyme genes in *E. coli* (Module 2_3) and *E. coli* (Module 1_2_3).


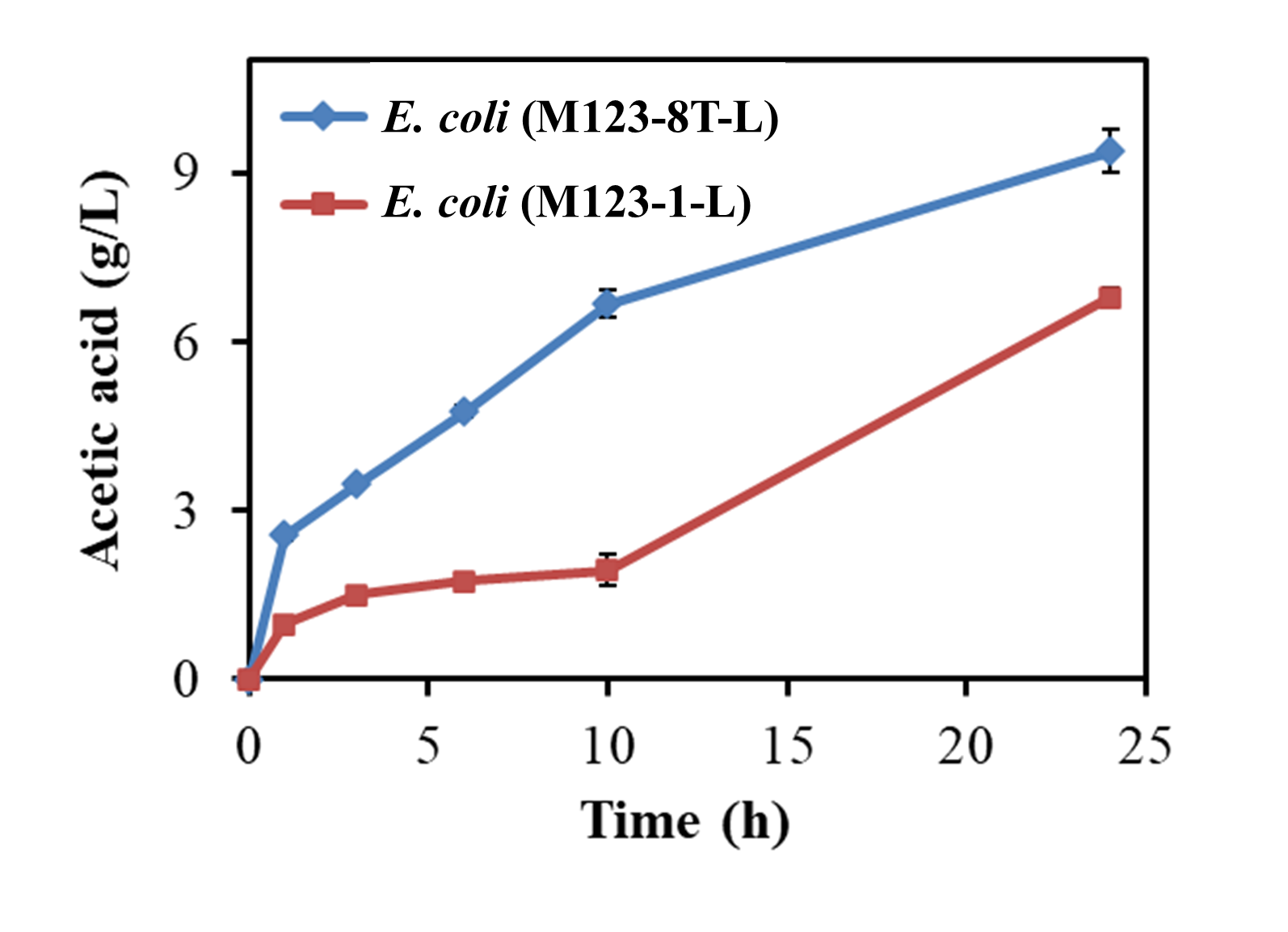


# Figure S6. Analysis of acetic acid. Reaction conditions: *E. coli* (M123-8T-L) and *E. coli* (M123-1-L) were resuspended in phosphate buffer (pH 8.0, 100 mM) at a cell density of 16 g CDW L^-1^, 5% glucose, reactions were performed at 25°C, 220 rpm. Average values are from triplicate experiments.


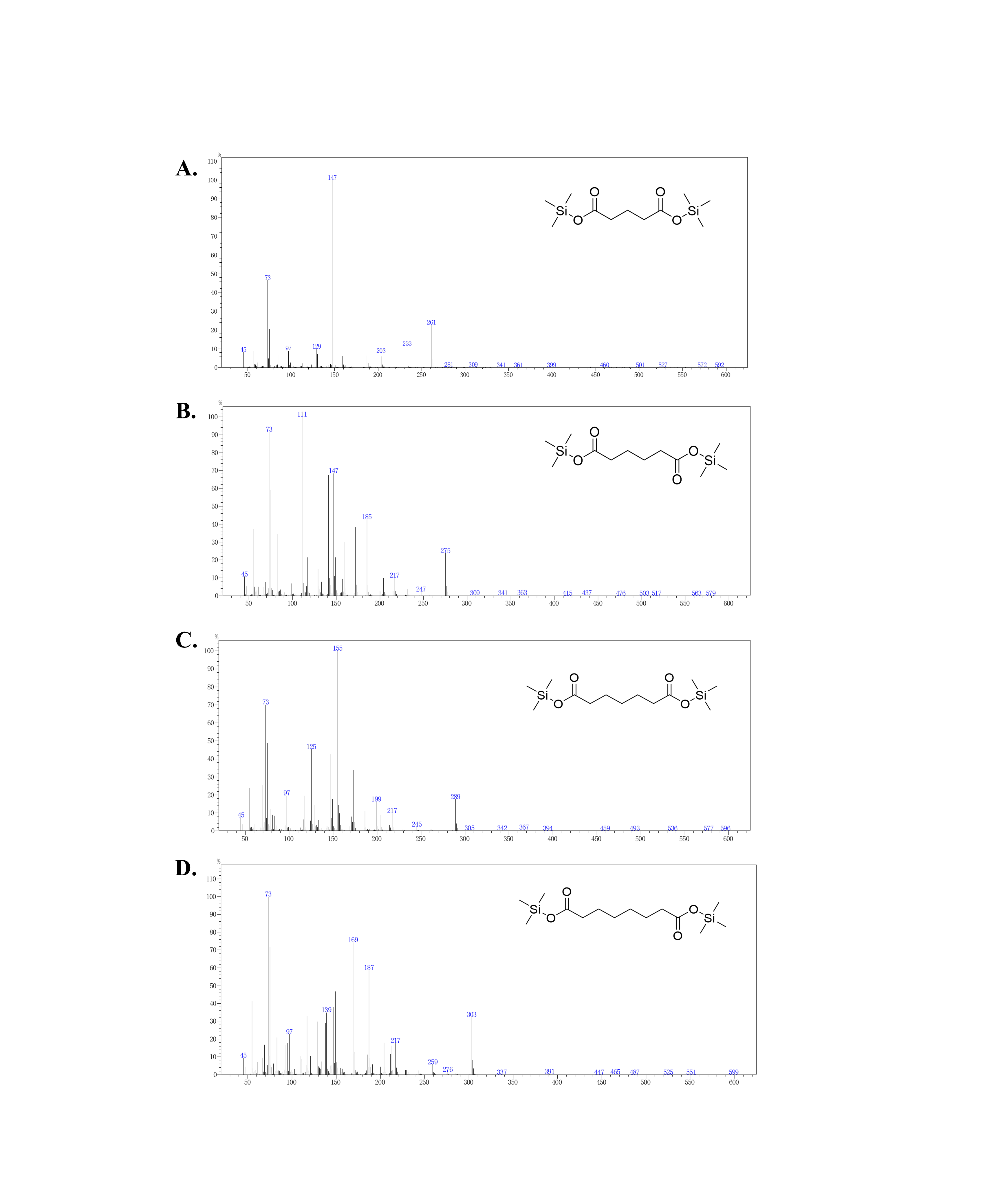


Figure S7. GC-MS analysis of α, ω-dicarboxylic acids derivatization. The fragmentation pattern was obtained for glutaric acid (**A**), adipic acid (**B**) pimelic acid (**C**), and suberic acid (**D**) after derivatization.

## Supplementary Nucleotide Sequence Information

| **P450 CHX**  ATGACCCAGACCGCCGCCGCTGCCGGTCGTGCAGATGCTTATAGCATTCCGCTGGATAAAATTAATGTTGCAGATCCGGCACTGTTTCGCGATGATACCGTTTGGCCGTATTTTGAACGTCTGCGCAAAGAAGATCCGATTCATTATTGTAAAGAGAGTCAGTATGGTCCGTATTGGAGTGTTACCAAATTTAAAGATATCATGAGCGTGGATACCAATCATAAAGTTTTTAGTGCCGAAGCACGTGATGGTGGCATTACCATTCTGGATAATAATGCAGCCGTTAGCCTGCCGATGTTTATTGCAATGGATCCGCCGAAACATGATGTGCAGCGTAAAGCCGTTAGCCCGATTTTTGCCCAGGAAAATCTGGCCAAACTGGAAGGTCTGATTCGCCAGCGCACCCAGAATGCACTGGATGCCCTGCCGATTGGCGAAACCTTTAATTGGGTTGAACGCGTGAGTATTAATCTGACCACCCAGATGCTGGCAACCCTGTTTGGTTTTCCGTTTGAAGAACGCACCAAACTGACCCGCTGGAGTGATATTGCCACCTGTGAACTGGGTACCTTTGGCATTGAAACCGAAGAACAGCGTATTAAAGAATTTCAGGAATGTGGTGCATATATGAGTCGTCTGTTTAATGAACGTGCAAAAGCAGATCCGCAGCCGGATCTGCTGAGTATGCTGGCACATAGTGAAAGCACCCGTAATATGACCCCGGAAGAATTTATGGGTAATATGGTGCTGCTGGTTGTGGGTGGTAATGATACCACCCGTAATAGCATGAGTGGCGGCGTGCTGGCCCTGAATCAGTATCCGGAAGAATATCGCAAACTGTGCGCCAATCCGGCCCTGATTCCGAGCATGGTGAGTGAAATTATTCGCTGGCAGACCCCGCTGAGTCATATTCGCCGTACCGCACTGCAGGATTTTGAACTGGGCGGCAAACAGATTAAAAAAGGTGATAAAGTTGTGATGTGGTATGCAAGTGGCAATCGTGATGAAGAAGCCATTGAAAATGCCAATCAGTTTATTATTGACCGTGCAAATCCGCGCCAGCATCTGAGCTTTGGCTTTGGCATTCATCGTTGCGTTGGTAATCGCCTGGCAGAAATGCAGCTGCGTGTTCTGTGGGAAGAAGCACTGAAACGTTGGCCGAAACCGGGCCAGATTGAAGTGGTTGGTGAACCGCAGCGTTATTTTAGTCCGCTGATGCGCGGCTTTGAAGCCCTGCCGGTTCGCATTAATGCCTAA |
| --- |
| **LbADH**  ATGAGCAATCGTCTGGATGGTAAAGTTGCAATTATTACCGGTGGCACCTTAGGTATTGGTCTGGCAATTGCAACCAAATTTGTTGAAGAGGGTGCCAAAGTTATGATTACCGGTCGTCATAGTGATGTTGGTGAAAAAGCAGCAAAAAGCGTTGGTACACCGGATCAGATTCAGTTTTTTCAGCATGATAGCAGTGATGAAGATGGTTGGACCAAACTGTTTGATGCAACCGAAAAAGCATTTGGTCCGGTTAGCACCCTGGTTAATAATGCAGGTATTGCAGTGAATAAGAGCGTTGAAGAAACCACCACCGCAGAATGGCGTAAACTGCTGGCAGTTAATCTGGATGGCGTTTTTTTTGGTACACGTCTGGGTATTCAGCGCATGAAAAACAAAGGTCTGGGTGCAAGCATTATCAACATGAGCAGCATTGAAGGTTTTGTTGGTGATCCGAGCCTGGGTGCATATAATGCAAGCAAAGGTGCAGTTCGTATTATGAGCAAAAGCGCAGCACTGGATTGTGCACTGAAAGATTATGATGTTCGTGTGAATACCGTTCATCCGGGTTATATCAAAACACCGCTGGTTGATGATCTGCCTGGTGCCGAAGAAGCAATGAGCCAGCGTACAAAAACCCCGATGGGTCATATTGGTGAACCGAATGATATTGCCTATATCTGTGTTTATCTGGCCAGCAACGAAAGTAAATTTGCAACCGGTAGCGAATTTGTTGTGGATGGTGGTTATACCGCACAGTAA |
| **ChnA**  ATGGAAAAGATCATGAGCAACAAATTCAACAACAAAGTGGCACTGATTACCGGTGCAGGTAGCGGTATTGGTAAAAGCACCGCACTGCTGCTGGCACAGCAGGGTGTTAGCGTTGTTGTTAGCGATATTAATCTGGAAGCAGCCCAGAAAGTTGTGGATGAAATTGTTGCATTAGGTGGTAAAGCAGCAGCCAATAAAGCAAATACCGCAGAACCGGAAGATATGAAAGCAGCCGTGGAATTTGCAGTTAGCACCTTTGGTGCACTGCATCTGGCATTTAACAATGCAGGTATTCTGGGTGAAGTTAACAGCACCGAAGAACTGAGCATTGAAGGTTGGCGTCGTGTTATTGATGTTAATCTGAATGCCGTGTTCTACAGCATGCATTATGAAGTTCCGGCAATTCTGGCAGCCGGTGGTGGTGCAATTGTTAATACCGCAAGCATTGCAGGTCTGATTGGTATTCAGAATATTAGCGGTTATGTGGCAGCAAAACATGGTGTTACCGGTCTGACCAAAGCCGCAGCACTGGAATATGCAGATAAAGGTATTCGTATCAATAGCGTTCATCCGGGTTATATCAAAACACCGCTGATTGCAGAATTTGAAGAGGCCGAAATGGTTAAACTGCATCCGATTGGTCGTCTGGGTCAGCCGGAAGAAGTTGCACAGGTTGTTGCCTTTCTGCTGAGTGATGATGCAAGCTTTGTGACCGGTAGCCAGTATGTTGTTGATGGTGCATATACCAGCAAATAA |
| **Flag-BVMO**  atggattataaagatgatgatgataaatcacaaaaaatggattttgatgctatcgtgattggtggtggttttggcggactttatgcagtcaaaaaattaagagacgagctcgaacttaaggttcaggcttttgataaagccacggatgtcgcaggtacttggtactggaaccgttacccaggtgcattgacggatacagaaacccacctctactgctattcttgggataaagaattactacaatcgctagaaatcaagaaaaaatatgtgcaaggccctgatgtacgcaagtatttacagcaagtggctgaaaagcatgatttaaagaagagctatcaattcaataccgcggttcaatcggctcattacaacgaagcagatgccttgtgggaagtcaccactgaatatggtgataagtacacggcgcgtttcctcatcactgctttaggcttattgtctgcgcctaacttgccaaacatcaaaggcattaatcagtttaaaggtgagctgcatcataccagccgctggccagatgacgtaagttttgaaggtaaacgtgtcggcgtgattggtacgggttccaccggtgttcaggttattacggctgtggcacctctggctaaacacctcactgtcttccagcgttctgcacaatacagcgttccaattggcaatgatccactgtctgaagaagatgttaaaaagatcaaagacaattatgacaaaatttgggatggtgtatggaattcagcccttgcctttggcctgaatgaaagcacagtgccagcaatgagcgtatcagctgaagaacgcaaggcagtttttgaaaaggcatggcaaacaggtggcggtttccgtttcatgtttgaaactttcggtgatattgccaccaatatggaagccaatatcgaagcgcaaaatttcattaagggtaaaattgctgaaatcgtcaaagatccagccattgcacagaagcttatgccacaggatttgtatgcaaaacgtccgttgtgtgacagtggttactacaacacctttaaccgtgacaatgtccgtttagaagatgtgaaagccaatccgattgttgaaattaccgaaaacggtgtgaaactcgaaaatggcgatttcgttgaattagacatgctgataCTGgccacaggttttgatgccgtcgatggcaactatgtgcgcatggacattcaaggtaaaaacggcttggccATTaaagactactggaaagaaggtccgtcgagctatatgggtgtcaccgtaaataactatccaaacatgttcatggtgcttggaccgaatggcccgtttaccaacctgccgccatcaattgaatcacaggtggaatggatcagtgataccattcaatacacggttgaaaacaatgttgaatccattgaagcgacaaaagaagcggaagaacaatggactcaaacttgcgccaatattgcggaaatgaccttattccctaaagcgcaatcctggatttttggtgcgaatatcccgggcaagaaaaacacggtttacttctatctcggtggtttaaaagaatatcgcagtgcgctagccaactgcaaaaaccatgcctatgaaggttttgatattcaattacaacgttcagatatcaagcaacctgccaatgcctaa |
| **ChnD**  ATGCATTGTTATTGCGTTACCCATCATGGTCAGCCGCTGGAAGATGTTGAAAAAGAAATTCCGCAGCCGAAAGGCACCGAAGTTCTGCTGCATGTTAAAGCAGCAGGTCTGTGTCATACCGATCTGCATCTGTGGGAAGGTTATTATGATTTAGGTGGTGGTAAACGTCTGAGCCTGGCAGATCGTGGTCTGAAACCGCCTCTGACACTGAGCCATGAAATTACCGGTCAGGTTGTTGCAGTTGGTCCGGATGCAGAAAGCGTTAAAGTTGGTATGGTTAGCCTGGTTCATCCGTGGATTGGTTGTGGTGAATGTAATTATTGTAAACGCGGTGAAGAAAACCTGTGTGCAAAACCGCAGCAGCTGGGTATTGCAAAACCTGGTGGTTTTGCAGAATACATTATTGTTCCGCATCCGCGTTATCTGGTTGATATTGCAGGTCTGGATCTGGCCGAAGCAGCACCGCTGGCATGTGCCGGTGTTACCACCTATAGCGCACTGAAAAAATTCGGTGATCTGATTCAGAGCGAACCGGTTGTTATTATTGGTGCCGGTGGTCTGGGTCTGATGGCACTGGAACTGCTGAAAGCAATGCAGGCAAAAGGTGCAATTGTTGTGGATATCGATGATAGCAAACTGGAAGCAGCCCGTGCAGCCGGTGCACTGAGCGTGATTAATAGCCGTAGCGAAGATGCAGCACAGCAGCTGATTCAGGCCACCGATGGTGGTGCACGTCTGATTCTGGACCTGGTTGGTAGCAATCCGACACTGAGTCTGGCACTGGCAAGCGCAGCACGTGGTGGTCATATTGTTATTTGTGGCCTGATGGGTGGTGAAATCAAACTGAGCATTCCGGTTATTCCGATGCGTCCGCTGACCATTCAGGGTAGCTATGTTGGCACCGTTGAAGAACTGCGTGAACTGGTTGAGCTGGTTAAAGAAACCCATATGAGCGCAATTCCGGTGAAAAAACTGCCGATTAGCCAGATTAATAGTGCCTTTGGCGATCTGAAAGATGGTAATGTTATTGGTCGTATCGTTCTGATGCACGAGAACTAA |
| **ChnE**  ATGAACTATCCGAATATTCCGCTGTATATTAACGGCGAATTTCTGGATCATACCAATCGTGATGTGAAAGAAGTGTTTAACCCGGTTAACCATGAATGCATTGGTCTGATGGCATGTGCAAGCCAGGCAGATCTGGATTATGCACTGGAAAGCAGCCAGCAGGCATTTCTGCGTTGGAAAAAAACCAGTCCGATTACACGTAGCGAAATTCTGCGTACCTTTGCAAAACTGGCACGTGAAAAAGCAGCAGAAATTGGTCGCAATATTACCCTGGATCAGGGCAAACCGCTGAAAGAAGCAATTGCCGAAGTTACCGTTTGTGCAGAACATGCAGAATGGCATGCAGAAGAATGTCGTCGTATTTATGGTCGTGTTATTCCGCCTCGTAATCCGAATGTTCAGCAGCTGGTTGTTCGTGAACCGCTGGGTGTTTGTCTGGCATTTAGCCCGTGGAATTTTCCGTTTAATCAGGCCATTCGTAAAATCAGCGCAGCAATTGCAGCAGGTTGTACCATTATTGTTAAAGGTAGCGGTGATACCCCGAGCGCAGTTTATGCAATTGCCCAGCTGTTTCATGAAGCAGGTCTGCCGAATGGTGTTCTGAATGTTATTTGGGGTGATAGCAACTTCATCAGCGACTATATGATTAAAAGCCCGATCATCCAGAAAATCAGCTTTACCGGTAGCACACCGGTTGGTAAAAAACTGGCCAGCCAGGCAAGCCTGTATATGAAACCGTGTACCATGGAATTAGGTGGTCATGCACCGGTTATTGTTTGTGATGATGCAGATATTGATGCAGCCGTTGAACATCTGGTTGGTTACAAATTTCGTAATGCAGGTCAGGTTTGTGTTAGCCCGACACGTTTTTATGTTCAAGAGGGCATCTATAAAGAGTTTAGCGAAAAAGTTGTTCTGCGTGCCAAGCAGATTAAAGTTGGTTGTGGTCTGGATGCAAGCAGCGATATGGGTCCGCTGGCACAGGCACGTCGTATGCATGCAATGCAGCAGATCGTTGAAGATGCAGTTCATAAAGGTAGTAAACTGCTGTTAGGTGGCAACAAGATTAGCGATAAAGGCAACTTTTTTGAACCGACCGTTCTGGGTGATCTGTGTAATGATACCCAGTTTATGAACGATGAACCGTTTGGTCCGATTATCGGTCTGATTCCGTTTGATACCATTGATCATGTTCTGGAAGAAGCAAATCGTCTGCCGTTTGGCCTGGCAAGCTATGCATTTACCACCAGTAGCAAAAATGCACACCAGATTAGCTATGGTCTGGAAGCAGGTATGGTTAGCATTAACCATATGGGTTTAGCACTGGCAGAAACCCCGTTTGGTGGTATTAAAGATAGTGGTTTTGGTAGCGAAGGTGGCATTGAAACCTTTGATGGTTATCTGCGCACCAAATTTATCACCCAGCTGAACTAA |
| **Lactonase**  ATGACCAATATTAGCGAAACCCTGAGCACCGCACCTGGTGGTGCAGCAGGTCCGGATGTTCTGCGTGATCTGTATGCAGATTGGAGCGAAATTATGGCAGCAACACCGGATCTGACCATTCGTCTGCTGCGTAGCCTGTTTGATGAATGGCATCAGCCGACCGTTGAACCGGAAGGTGTTACCTATCGTGAAGAAACCGTTGGTGGTGTTCCTGGTATTTGGTGTCTGCCGCAGGGTGCAGATGGTAGCAAAGTTCTGCTGTATACCCATGGTGGTGGTTTTGCAGTTGGTAGCGCAGCAAGCCATCGTAAACTGGCAGGTCATGTTGCAAAAGCACTGGGTGCCGTTGGTTTTGTTCTGGATTATCGTCGTGCACCGGAATTTCAGCATCCGGCACAGATTGAAGATGGTGTTGCAGCATTTGATGCACTGGTTGCAAATGGTATTGCACCGCAGGATATTACCACCATTGGTGATAGTGCCGGTGGTAATCTGGCAGTTGCAATTGCCCTGAGCCTGCGTGAACAGGGTAAACAAGGTCCGGGTAGCGTTATTGCATTTAGCCCGTGGCTGGATATGGAAAATAAAGGTGAAACCCTGGCCACCAATAATGATACCGATGCACTGATTACACCGGAACTGCTGGAAGGCATGATTGCCGGTGTGCTGGGTGATACCATTGATCCGAAAACACCGCTGGCAAATCCGCTGTATGCCGATTTTACCGGTTTTCCGCGTCTGTATATCACCGCAGGTAGCGTTGAAAGCCTGCTGGATAATGCAACCCGTCTGGAAAAATTAGCAGCATCTGCCGGTGTTGATGTTACCCTGAGTATTGGTGAAGGTCAGCAGCATGTTTATCCGTTTCTGGCAGGCCGTAGCGCACTGGTGGATGATGAATTTGCAAAGCTGGCAGCATGGTATCAGAAAGCCCTGGAATAA |
| **Fdr**  ATGTTGAATGCGAGTGTGGCTGGCGGAGCAGCTACCACCACCTATGGCAACCGGCTCTTTATCTATGAAGTGATCGGTCTGCGCCAAGCCGAGGGCGAACCGTCCGACAGCTCAATCCGCCGTAGTGGCAGCACCTTCTTCAAGGTGCCTTACAGCCGGATGAATCAAGAAATGCAACGGATTTTGCGCCTTGGCGGCAAAATCGTTAGCATCCGGCCTGCGGAGGAAGCAGCCGCGAATAATGGTGCGGCTCCTCTACAGGCAGCAGCTGAAGAACCTGCTGCAGCACCAACCCCCGCTCCGGCTGCCAAAAAACATTCAGCCGAAGACGTGCCTGTCAATATCTACCGGCCTAACAAGCCTTTCGTAGGCAAGGTGCTCTCGAACGAGCCCTTGGTTCAAGAAGGCGGGATTGGTGTTGTGCAGCACCTCACCTTCGATATTTCGGAAGGCGATCTGCGCTACATCGAAGGTCAAAGTATCGGGATTATCCCGGATGGCACCGATGACAAAGGCAAGCCGCACAAGCTCCGTCTTTACTCGATCGCATCCACTCGCCACGGCGACCACGTGGATGACAAAACCGTCTCGCTGTGCGTGCGCCAGCTGCAGTACCAGAACGAAGCCGGCGAAACGATTAATGGCGTCTGCTCGACTTTCCTCTGTGGTCTGAAGCCAGGCGATGACGTCAAGATCACCGGTCCTGTGGGCAAAGAAATGCTCCTACCGGCGGACACAGACGCCAACGTGATCATGATGGGTACTGGCACCGGGATTGCTCCGTTCCGAGCCTACCTATGGCGGATGTTTAAAGACAACGAGCGAGCCATCAACAGCGAGTATCAATTCAACGGCAAGGCTTGGTTGATCTTCGGGATTCCGACGACCGCCAACATCCTCTACAAAGAGGAGCTGGAAGCGCTGCAGGCTCAGTATCCAGATAACTTCCGCCTGACCTACGCGATCAGCCGCGAGCAGAAAAATGAAGCGGGCGGCCGGATGTACATCCAAGACCGCGTCGCTGAACATGCTGACGAGATCTGGAACCTACTCAAGGACGAAAAAACCCACGTCTATATCTGTGGTTTGCGTGGCATGGAAGATGGGATCGATCAAGCCATGACCGTCGCAGCTGCCAAGGAAGATGTGGTTTGGTCTGACTACCAACGCACCCTCAAGAAAGCGGGTCGTTGGCATGTTGAAACCTACTAG |
| **Fdx**  ATGGCAACCTACAAGGTTACGCTCGTCAATGCTGCCGAAGGCTTGAACACCACGATCGACGTGGCTGACGATACCTACATCTTGGACGCCGCTGAAGAGCAAGGCATTGACCTGCCTTACTCCTGCCGTGCTGGTGCTTGCTCGACCTGTGCTGGCAAAGTCGTCTCTGGTACCGTCGACCAATCGGATCAATCCTTCTTGGATGACGACCAAATTGCAGCAGGCTTTGTCCTGACCTGCGTCGCCTATCCGACCTCCGATGTGACGATCGAAACCCACAAAGAAGAAGACCTCTACTAG |
| **Fpr**  ATGGCTGATTGGGTAACAGGCAAAGTCACTAAAGTGCAGAACTGGACCGACGCCCTGTTTAGTCTCACCGTTCACGCCCCCGTGCTTCCGTTTACCGCCGGGCAATTTACCAAGCTTGGCCTTGAAATCGACGGCGAACGCGTCCAGCGCGCCTACTCCTATGTAAACTCGCCCGATAATCCCGATCTGGAGTTTTACCTGGTCACCGTCCCCGATGGCAAATTAAGCCCACGACTGGCGGCACTGAAACCAGGCGATGAAGTGCAGGTGGTTAGCGAAGCGGCAGGATTCTTTGTGCTCGATGAAGTGCCGCACTGCGAAACGCTATGGATGCTGGCAACCGGTACAGCGATTGGCCCTTATTTATCGATTCTGCAACTAGGTAAAGATTTAGATCGCTTCAAAAATCTGGTCCTGGTGCACGCCGCACGTTATGCCGCCGACTTAAGCTATTTGCCACTGATGCAGGAACTGGAAAAACGCTACGAAGGAAAACTGCGCATTCAGACGGTGGTCAGTCGGGAAACGGCAGCGGGGTCGCTCACCGGACGGATACCGGCATTAATTGAAAGTGGGGAACTGGAAAGCACGATTGGCCTGCCGATGAATAAAGAAACCAGCCATGTGATGCTGTGCGGCAATCCACAGATGGTGCGCGATACACAACAGTTGCTGAAAGAGACCCGGCAGATGACGAAACATTTACGTCGCCGACCGGGCCATATGACAGCGGAGCATTACTGGTAA |
| **YkuN**  ATGGCTAAAGCCTTGATTACATATGCCAGCATGTCAGGAAATACAGAAGACATTGCCTTCATAATAAAAGATACGCTTCAGGAATATGAGTTGGATATCGATTGTGTCGAGATAAATGATATGGATGCGTCTTGTTTAACCTCCTATGATTATGTACTGATTGGCACCTATACATGGGGGGACGGCGATTTGCCCTACGAAGCGGAGGATTTTTTCGAAGAGGTCAAACAGATTCAGCTTAATGGTTTAAAAACAGCCTGCTTCGGGTCTGGCGATTATTCTTATCCAAAGTTTTGCGAAGCGGTGAATTTGTTCAATGTCATGCTGCAAGAGGCGGGAGCTGCTGTTTACCAGGAAACACTAAAAATTGAATTAGCGCCTGAAACAGATGAAGATGTGGAAAGCTGCCGAGCGTTTGCGAGAGGTTTTCTTGCATGGGCAGATTATATGAACAAGGAAAAAATCCATGTTTCATAA |
| **CamA**  ATGGTTAACGCCAATGATAATGTTGTTATCGTTGGTACCGGTCTGGCCGGCGTTGAAGTGGCCTTTGGCCTGCGCGCAAGTGGCTGGGAAGGCAATATTCGTCTGGTTGGTGATGCCACCGTGATTCCGCATCATCTGCCGCCGCTGAGCAAAGCATATCTGGCAGGCAAAGCCACCGCCGAAAGTCTGTATCTGCGTACCCCGGATGCATATGCAGCACAGAATATTCAGCTGCTGGGCGGTACCCAGGTTACCGCCATTAATCGCGATCGTCAGCAGGTGATTCTGAGTGATGGCCGCGCACTGGATTATGATCGCCTGGTGCTGGCAACCGGCGGCCGTCCTCGTCCTCTGCCTGTGGCAAGTGGCGCAGTTGGTAAAGCCAATAATTTTCGTTATCTGCGCACCCTGGAAGATGCAGAATGTATTCGTCGTCAGCTGATTGCAGATAATCGTCTGGTTGTTATTGGTGGTGGCTATATTGGCCTGGAAGTTGCCGCAACCGCCATTAAAGCCAATATGCATGTGACCCTGCTGGATACCGCCGCCCGCGTGCTGGAACGTGTGACCGCTCCGCCGGTTAGTGCCTTTTATGAACATCTGCATCGCGAAGCCGGCGTTGATATTCGCACCGGCACCCAGGTTTGCGGTTTTGAAATGAGCACCGATCAGCAGAAAGTTACCGCCGTTCTGTGCGAAGATGGCACCCGCCTGCCGGCAGATCTGGTTATTGCCGGCATTGGTCTGATTCCGAATTGTGAACTGGCCAGTGCCGCCGGCCTGCAGGTTGATAATGGCATTGTTATTAATGAACACATGCAGACCAGTGATCCGCTGATTATGGCAGTGGGCGATTGTGCACGTTTTCATAGCCAGCTGTATGATCGCTGGGTTCGTATTGAAAGCGTGCCGAATGCCCTGGAACAGGCCCGTAAAATTGCAGCCATTCTGTGCGGTAAAGTGCCGCGCGATGAAGCCGCACCGTGGTTTTGGAGCGATCAGTATGAAATTGGTCTGAAAATGGTGGGTCTGAGTGAAGGTTATGATCGTATTATTGTGCGTGGTAGTCTGGCACAGCCGGATTTTAGCGTTTTTTATCTGCAGGGTGATCGTGTTCTGGCCGTGGATACCGTGAATCGTCCGGTGGAATTTAATCAGAGCAAACAGATTATTACCGATCGCCTGCCGGTGGAACCGAATCTGCTGGGCGATGAAAGCGTGCCTCTGAAAGAAATTATTGCCGCCGCAAAAGCCGAACTGAGTAGCGCCTAA |
| **CamB**  ATGAGCAAAGTGGTTTATGTTAGCCATGATGGCACCCGCCGTGAACTGGATGTGGCAGATGGTGTTAGCCTGATGCAGGCAGCAGTGAGCAATGGTATTTATGATATTGTGGGTGATTGTGGTGGTAGCGCAAGTTGTGCCACCTGTCATGTTTATGTGAATGAAGCATTTACCGATAAAGTGCCGGCAGCAAATGAACGCGAAATTGGTATGCTGGAATGCGTTACCGCAGAACTGAAACCGAATAGCCGCCTGTGCTGCCAGATTATTATGACCCCGGAACTGGATGGTATTGTGGTTGATGTGCCGGATCGTCAGTGGTAA |
| **116B46_**  CTGCGTCAGCCGGTGCGTATTGGTCCACCGCGTGCGAAAGACGTTGTGCGCACTATGGAAGTTGCTGCAGTTGAACGCCCGTCCGAAGATATCGTGGTTCTGCACCTGACCCGCCCGGACCGTCGTCCGCTGCCGCGTTGGTCTCCGGGCGCTCATATCGATATTGAATGTGGCGAACCTGATCGTTCCCGCCAGTATAGCCTGTGCTCTGACCCAGAAAACCGTGACGCATGGCGTGTAGCGGTACAGCGTGACCCGGCGAGCCGCGGCGGCTCTCGCTGGATTCACGAAGAGGTGCGTCCGGGTATGCTGCTGCGCGTTCGTGGTCCGCGTAATTCCTTCCGTCTGGACGAACACGCTCCGCGTTACCTGTTCCTGGCGGGTGGCATCGGCATCACCCCGATCATGACTATGGCGGCGCGCGCGAAAGAGCTGGGTACCGATTACGAACTGCATTATTCTGTGCGTTCTCGCACCAGCCTGATCTTCGTGGATGAACTGCGCCAGATCCACGGTGATCGCCTGCACGTGTACGTGAGCGAAGAAGGTGTGCGTAACGACCTGGCAGCGCTGATTCGCCGTGCGAGCGCTGGCACCCAGATCTACGCATGTGGCCCGCAGCGTATGCTGGACACCCTGGAACGCCTGATCGAAAACCGTCCGGAAGTAACCCTGCGTGTGGAACATTTCTTCGGCGAACCGAGCCACCTGGATCCGGCGAAAGAACGTCCGTTCCAGGTTGTGCTGCGTAACTCTGGTCTGACCGTTGAAGTTCCGGCCGATAAAACCCTGTTGGAAGTTTTGCGTGCATACAACATCGAAGTGCAGTCTGATTGTGAAGAAGGTCTGTGCGGCACTTGTGAAGTTTCTGTTGTTGAAGGTGAAGTTGATCACCGTGATTCCGTGCTGACCCGCGCAGAACGTCGTGAAAACCGTCGTATGATGTGCTGTTGCTCTCGTGCTAAAACCGAACGTCTGGTTCTGGACCTGTAA |
| **RhFRed_**  cggcatcaaccggtcaccatcggagaacccgccgcccgggcggtgtcccgcaccgtcaccgtcgagcgcctggaccggatcgccgacgacgtgctgcgcctcgtcctgcgcgacgccggcggaaagacattacccacgtggactcccggcgcccatatcgacctcgacctcggcgcgctgtcgcgccagtactccctgtgcggcgcgcccgatgcgccgagctacgagattgccgtgcacctggatcccgagagccgcggcggttcgcgctacatccacgaacagctcgaggtgggaagcccgctccggatgcgcggccctcggaaccatttcgcgctcgaccccggcgccgagcactacgtgttcgtcgccggcggcatcggcatcaccccagtcctggccatggccgaccacgcccgcgcccgggggtggagctacgaactgcactactgcggccgaaaccgttccggcatggcctatctcgagcgtgtcgccgggcacggtgaccgggccgccctgcacgtgtccgaggaaggcacccggatcgacctcgccgccctcctcgccgagcccgcccccggcgtccagatctacgcgtgcgggcccgggcggctgctcgccggactcgaggacgcgagccggaactggcccgacggggcgctgcacgtcgagcacttcacctcgtccctcgcggcgctcgatccggacgtcgagcacgccttcgacctcgaactgcgtgactcggggctgaccgtgcgggtcgaacccacccagaccgtcctcgacgcgttgcgcgccaacaacatcgacgtgcccagcgactgcgaggaaggcctctgcggctcgtgcgaggtcgccgtcctcgacggcgaggtcgaccatcgcgacacggtgctgaccaaggccgagcgggcggcgaaccggcagatgatgacctgctgctcgcgtgcctgtggcgaccggctggccctgcgactctga |
| **19A12_**  GgtCAGTCTGCTAAAAAAGTACGCAAAAAGGCAGAAAACGCTCATAATACGCCGCTGCTTGTGCTATACGGTTCAAATATGGGAACAGCTGAAGGAACGGCGCGTGATTTAGCAGATATTGCAATGAGCAAAGGATTTGCACCGCAGGTCGCAACGCTTGATTCACACGCCGGAAATCTTCCGCGCGAAGGAGCTGTATTAATTGTAACGGCGTCTTATAACGGTCATCCGCCTGATAACGCAAAGCAATTTGTCGACTGGTTAGACCAAGCGTCTGCTGATGAAGTAAAAGGCGTTCGCTACTCCGTATTTGGATGCGGCGATAAAAACTGGGCTACTACGTATCAAAAAGTGCCTGCTTTTATCGATGAAACGCTTGCCGCTAAAGGGGCAGAAAACATCGCTGACCGCGGTGAAGCAGATGCAAGCGACGACTTTGAAGGCACATATGAAGAATGGCGTGAACATATGTGGAGTGACGTAGCAGCCTACTTTAACCTCGACATTGAAAACAGTGAAGATAATAAATCTACTCTTTCACTTCAATTTGTCGACAGCGCCGCGGATATGCCGCTTGCGAAAATGCACGGTGCGTTTTCAACGAACGTCGTAGCAAGCAAAGAACTTCAACAGCCAGGCAGTGCACGAAGCACGCGACATCTTGAAATTGAACTTCCAAAAGAAGCTTCTTATCAAGAAGGAGATCATTTAGGTGTTATTCCTCGCAACTATGAAGGAATAGTAAACCGTGTAACAGCAAGGTTCGGCCTAGATGCATCACAGCAAATCCGTCTGGAAGCAGAAGAAGAAAAATTAGCTCATTTGCCACTCGCTAAAACAGTATCCGTAGAAGAGCTTCTGCAATACGTGGAGCTTCAAGATCCTGTTACGCGCACGCAGCTTCGCGCAATGGCTGCTAAAACGGTCTGCCCGCCGCATAAAGTAGAGCTTGAAGCCTTGCTTGAAAAGCAAGCCTACAAAGAACAAGTGCTGGCAAAACGTTTAACAATGCTTGAACTGCTTGAAAAATACCCGGCGTGTGAAATGAAATTCAGCGAATTTATCGCCCTTCTGCCAAGCATACGCCCGCGCTATTACTCGATTTCTTCATCACCTCGTGTCGATGAAAAACAAGCAAGCATCACGGTCAGCGTTGTCTCAGGAGAAGCGTGGAGCGGATATGGAGAATATAAAGGAATTGCGTCGAACTATCTTGCCGAGCTGCAAGAAGGAGATACGATTACGTGCTTTATTTCCACACCGCAGTCAGAATTTACGCTGCCAAAAGACCCTGAAACGCCGCTTATCATGGTCGGACCGGGAACAGGCGTCGCGCCGTTTAGAGGCTTTGTGCAGGCGCGCAAACAGCTAAAAGAACAAGGACAGTCACTTGGAGAAGCACATTTATACTTCGGCTGCCGTTCACCTCATGAAGACTATCTGTATCAAGAAGAGCTTGAAAACGCCCAAAGCGAAGGCATCATTACGCTTCATACCGCTTTTTCTCGCATGCCAAATCAGCCGAAAACATACGTTCAGCACGTAATGGAACAAGACGGCAAGAAATTGATTGAACTTCTTGATCAAGGAGCGCACTTCTATATTTGCGGAGACGGAAGCCAAATGGCACCTGCCGTTGAAGCAACGCTTATGAAAAGCTATGCTGACGTTCACCAAGTGAGTGAAGCAGACGCTCGCTTATGGCTGCAGCAGCTAGAAGAAAAAGGCCGATACGCAAAAGACGTGTGGGCTGGGTAA |
| **LkADH（Lentilactobacillus kefirri）**  ATGACTGATCGTTTAAAAGGCAAAGTAGCAATTGTAACTGGCGGTACCTTGGGAATTGGCTTGGCAATCGCTGATAAGTTTGTTGAAGAAGGCGCAAAGGTTGTTATTACCGGCCGTCACGCTGATGTAGGTGAAAAAGCTGCCAAATCAATCGGCGGCACAGACGTTATCCGTTTTGTCCAACACGATGCTTCTGATGAAGCCGGCTGGACTAAGTTGTTTGATACGACTGAAGAAGCATTTGGCCCAGTTACCACGGTTGTCAACAATGCCGGAATTGCGGTCAGCAAGAGTGTTGAAGATACCACAACTGAAGAATGGCGCAAGCTGCTCTCAGTTAACTTGGATGGTGTCTTCTTCGGTACCCGTCTTGGAATCCAACGTATGAAGAATAAAGGACTCGGAGCATCAATCATCAATATGTCATCTATCGAAGGTTTTGTTGGTGATCCAACTCTGGGTGCATACAACGCTTCAAAAGGTGCTGTCAGAATTATGTCTAAATCAGCTGCCTTGGATTGCGCTTTGAAGGACTACGATGTTCGGGTTAACACTGTTCATCCAGGTTATATCAAGACACCATTGGTTGACGATCTTGAAGGGGCAGAAGAAATGATGTCACAGCGGACCAAGACACCAATGGGTCATATCGGTGAACCTAACGATATCGCTTGGATCTGTGTTTACCTGGCATCTGACGAATCTAAATTTGCCACTGGTGCAGAATTCGTTGTCGATGGTGGATACACTGCTCAATAA |
| **TbADH**  ATGAAAGGTTTTGCAATGCTGAGTATTGGTAAAGTTGGCTGGATCGAAAAAGAAAAACCGGCACCGGGTCCGTTTGATGCAATTGTTCGTCCGCTGGCAGTTGCACCGTGTACCAGCGATATTCATACCGTTTTTGAAGGTGCAATTGGCGAACGCCATAATATGATTCTGGGTCATGAAGCAGTTGGTGAAGTTGTTGAAGTGGGTAGCGAAGTGAAAGATTTTAAACCGGGTGATCGTGTTGTTGTTCCGGCAATTACACCGGATTGGCGTACCAGTGAAGTTCAGCGTGGTTATCATCAGCATAGCGGTGGTATGCTGGCAGGTTGGAAATTTAGCAATGTTAAAGATGGTGTGTTCGGCGAATTTTTCCATGTTAATGATGCCGATATGAATCTGGCACATCTGCCGAAAGAAATTCCGCTGGAAGCAGCAGTTATGATTCCGGATATGATGACCACCGGTTTTCATGGTGCAGAACTGGCAGATATTGAACTGGGTGCAACCGTTGCAGTTTTAGGTATTGGTCCGGTTGGTCTGATGGCCGTTGCCGGTGCAAAACTGCGTGGTGCAGGTCGTATTATTGCAGTTGGTAGCCGTCCGGTTTGTGTTGATGCAGCAAAATACTATGGTGCCACCGATATCGTGAATTACAAAGATGGTCCGATTGAAAGCCAGATTATGAACCTGACCGAAGGTAAAGGTGTGGATGCAGCCATTATTGCCGGTGGTAATGCAGATATCATGGCAACCGCAGTTAAAATTGTTAAACCTGGTGGTACAATTGCCAACGTGAACTATTTTGGTGAAGGTGAAGTTCTGCCGGTTCCGCGTCTGGAATGGGGTTGTGGTATGGCACATAAAACCATTAAAGGTGGTCTGTGTCCTGGTGGTCGTCTGCGTATGGAACGTCTGATTGATCTGGTTTTCTATAAACGTGTTGATCCGAGCAAACTGGTTACCCATGTTTTTCGTGGCTTTGACAATATCGAGAAAGCATTCATGCTTATGAAAGACAAACCGAAGGATCTGATTAAACCGGTTGTTATTCTGGCCTAA |
| **TmCHMO**  ATGAGCACCACACAGACACCGGATCTGGATGCAATTGTTATTGGTGCAGGTTTTGGTGGCATTTACATGCTGCATAAACTGCGTAATGATCTGGGTCTGAGCGTTCGTGTTTTTGAAAAAGGTGGTGGTGTTGGTGGCACCTGGTATTGGAACAAATATCCGGGTGCAAAAAGCGATACCGAAGGTTTTGTTTATCGCTACAGCTTCGATAAAGAACTGCTGCGTGAATATGATTGGACCACACGTTATCTGGATCAGCCGGATGTTCTGGCATATCTGGAACATGTTGTTGAACGTTATGATCTGGCACGTGATATCCAGCTGAATACCGAAGTTACCGATGCCATTTTTGATGAAGAAACCGAACTGTGGCGTGTTACCACCGCAGGCGGTGAAACCCTGACCGCACGTTTTCTGGTTACCGCACTGGGTCTGCTGAGCCGTAGCAACATTCCGGATATTCCGGGTCGTGATAGCTTTGCAGGTCGTCTGGTTCATACCAATGCATGGCCTGAAGATCTGGATATAACCGGTAAACGTGTTGGTGTGATTGGCACCGGTTCTACCGGTACACAGTTTATTGTTGCAGCAGCCAAAATGGCAGAACAGCTGACCGTTTTTCAGCGTACACCGCAGTATTGTGTTCCGAGCGGTAATGGTCCGATGGACCCGGATGAAGTTGCACGTATTAAACAGAACTTTGATAGCATCTGGGATCAAGTTCGTAGCAGCACCGTTGCATTTGGTTTTGAAGAAAGCACCGTGGAAGCAATGAGCGTTAGCGAAAGCGAACGTCAGCGTGTGTTTCAGCAGGCATGGGATAAAGGTAATGGTTTTCGTTTTATGTTTGGCACCTTTTGCGATATTGCAACCAATCCGGAAGCAAATGCAGCAGCAGCCGCATTTATTCGTAGCAAAATTGCCGAAATTGTGAAAGATCCGGAAACCGCACGTAAACTGACCCCGACCGATCTGTATGCAAAACGTCCGCTGTGTAATGAAGGTTATTATGAAACCTATAACCGCGATAATGTTAGCCTGGTGAGCCTGAAAGAAACCCCGATTGAAGAAATTGTTCCGCAGGGTGTTCGTACCAGTGATGGTGTTGTGCATGAACTGGATGTGCTGGTTTTTGCAACCGGTTTTGATGCAGTTGATGGTAATTATCGTGCAATGAATCTGCGTGGTCGTGATGGTCGTCATATTAATGAACATTGGACCGAAGGTCCGACCAGCTATCTGGGTGTTACCAAAGCAGGTTTTCCGAACATGTTTATGATTCTGGGTCCGAATGGTCCGTTTACCAATCTGCCTCCGAGCATTGAAGCACAGGTTGAATGGATTAGCGATCTGATTGATAAAGCAACCCGTGAAGGTCTGACCACCGTTGAACCGACCGCAGATGCAGAACGTGAATGGACCGAAACCTGTGCAGAAATTGCAAATATGACCCTGTTTCCGAAAGCCGATAGCTGGATTTTTGGTGCAAATATTCCTGGTAAACGTCACGCCGTTATGTTTTATCTTGGTGGTCTGGGCAATTATCGTCGTCAGCTGGCAGATGTTGCAGATGGTGGTTATCGTGGTTTTCAGCTGCGTGGTGAACGTGCACAGGCAGTTGCATAA |
